# Impact of the early-life skin microbiota on the development of canine atopic dermatitis in a high-risk breed birth cohort

**DOI:** 10.1038/s41598-020-57798-x

**Published:** 2020-01-23

**Authors:** S. Rodriguez-Campos, A. Rostaher, L. Zwickl, N. Fischer, I. Brodard, S. Vidal, B. W. Brandt, C. Favrot, V. Perreten

**Affiliations:** 1 Institute of Veterinary Bacteriology, Vetsuisse Faculty, University of Bern, Länggassstrasse 122, CH-3012 Bern, Switzerland; 2 Bacteriology and Mycology Unit, Faculty of Veterinary Medicine, Norwegian University of Life Sciences, Ullevålsveien 72, 0454 Oslo, Norway; 3 Clinic for Small Animal Internal Medicine, Vetsuisse Faculty, University of Zurich, Winterthurerstrasse 260, 8057 Zurich, Switzerland; 4 Present Address: Gnubiotics Sciences SA, Microbiome Research, StartLab/Biopôle, Bat SE-B, Route de la Corniche 5, 1066 Epalinges, Switzerland; 5 Department of Preventive Dentistry, Academic Centre for Dentistry Amsterdam (ACTA), University of Amsterdam and Vrije Universiteit Amsterdam, Gustav Mahlerlaan 3004, 1081 LA Amsterdam, The Netherlands

**Keywords:** Metagenomics, Fungi

## Abstract

Canine atopic dermatitis (CAD) is a prevalent inflammatory skin disease of dogs worldwide. Certain breeds such as the West Highland White Terriers (WHWT) are predisposed to suffer from CAD. Microbial dysbiosis is known to play a significant role in the pathogenesis of the disease, which is similar to its human counterpart, atopic dermatitis (AD). To date, no large cohort-study has been conducted in a predisposed dog breed to study the impact of the early-life microbiota on the development of CAD, as well as the possible implication of factors such as hygiene and access to the outdoors. In this study skin samples of 143 WHWT, including 109 puppies up to three weeks old and 34 parent dogs, from 17 breeders, were subjected to 16S rRNA gene and ITS2 amplicon sequencing to disclose the bacterial and fungal oral and skin microbiota, respectively. The oral samples served as a control group to confirm differences between haired and mucosal surfaces. The cutaneous microbiota differed between sample sites and age of the dogs. The season of sampling, geographical origin as well as hygiene status of the household and the access to the outdoors shaped the skin microbiota of the puppies significantly. However, we found that the individual early-life microbiota did not predispose for the later development of CAD.

## Introduction

Among the inflammatory skin diseases occurring in dogs, canine atopic dermatitis (CAD) is prevalent with up to 10% of dogs affected worldwide^1,2^. CAD is a genetically predisposed inflammatory and pruritic allergic skin disease with characteristic clinical features associated with IgE antibodies most commonly directed against environmental allergens^3^. Across species, similarities in the clinical manifestations of atopic dermatitis have been observed and the disease is becoming more common^4^. In contrast to humans who may develop respiratory signs, dogs usually remain in the cutaneous stage of the disease^5^. Nevertheless, CAD is frequently discussed as a model for human atopic dermatitis (AD).

AD has a strong genetic component^6^, but its occurrence seems to be increasing in recent years due to environmental factors^2^. Among the discussions about protective and risk factors, the hygiene hypothesis^7^ has been prominent for many years. It speculates that early microbial exposure reduces the risk for developing allergies and, consequently, the improvement of hygiene may explain a rise in allergic diseases. This hypothesis has been challenged in more recent years and it was suggested to replace the term by “microbial exposure” or “microbial deprivation” hypothesis to avoid the misinterpretation of a good hygiene practice^8^. Along these lines, several factors in the early-life of humans that increase microbial exposure were described as protective, such as residence on a farm with livestock^9,10^.

Many of the environmental microbial factors associated with the increasing incidence of atopic dermatitis in humans^11^ can also be found in the environment of dogs^5,12^. Living in outdoor conditions and having contact with farm animals were, for instance, described as protective factors for CAD^13^, while high levels of exposure to tobacco smoke increased the rate of allergic skin disease in dogs^14^. Additionally, it is known that some dog breeds are more predisposed to develop CAD than others, underlining the genetic component of the disease^15,16^. West Highland White Terriers (WHWT) were among eleven breeds identified with a significantly increased odds ratio for CAD in a recent study from Australia^17^. Although exact data on the prevalence of CAD among dogs are missing, it is estimated to affect 30% of the dogs^4^ with the prevalence among predisposed breeds being higher. The most prominent bacterial genus implicated in AD is *Staphylococcu*s, and *S. aureus* and *S. pseudintermedius* were shown to aggravate clinical signs in humans^18^ and in dogs^19^, respectively. Fungi are thought to have less influence in the pathogenesis of AD. Yet, *Malassezia*, that has a commensal role in the healthy skin, may contribute to disease pathogenesis as shown in humans^20^ and dogs^21–25^.

Next-generation sequencing of hypervariable regions of the 16S rRNA gene of bacteria and of the internal transcribed spacer (ITS) region of fungi is nowadays frequently exploited to describe the microbial differences between healthy and allergic individuals^11,26,27^ and the changes in the microbiota during inflammatory processes and treatment^19,26^.

To date, no canine birth cohort study has been conducted to assess the impact of environmental factors and the mode of delivery on the later development of allergy. In this study, we aimed at comparing the cutaneous bacterial and fungal microbial profiles of cohabiting WHWTs from independent breeders. The allergy status of the enrolled puppies was assessed at adult age three years after sampling with the purpose of relating the early-life microbiota of the puppies to a possible later development of allergy.

## Results

### Data collection

Among the 17 breeders who participated in the study, nine classified with an intermediate hygiene status, six with a high and two with a low hygiene standard (Table 1). Seven breeders allowed the animals to have intermediate access (>1 to 3 hours per day) to the outdoor environment at the time of sampling; dogs from six breeders had > 3 hours of outdoor activity per day and dogs from four breeders only up to one hour (Table 1).

**Table 1:** Alphabetical code for the West Highland White Terrier breeders (= household), number and code of the litters per breeder, sampling year and season, collected data on hygiene status (1 = low standard, 2 = average standard, 3 = high standard), access to the outdoor environment of the puppies (1 = inside, 2 = inside with access to outdoor environment, 3 = outside) and number of puppies, bitches and stud dogs enrolled in the study. Bioregions are shown in Supplementary Fig. S3.

Household	Litter(s)	Sampling year/season	Bioregion^a^	Hygiene	Environment	No. of puppies	No. of adult bitches	No. of stud dogs
A	A1, A2	2014/spring	C.P.	1	2	17	4	
	A3, A4	2015/summer						
B	B1	2014/spring	C.P.	2	3	7	1	
C	C1	2014/summer	N.A.	3	3	5		
D	D1	2014/summer	C.P.	2	3	3	1	
E	E1	2014/summer	J.	3	2	17	5	
	E2	2015/winter						
	E3, E4	2015/summer						
	E5	2015/fall						
F	F1	2015/winter	C.P.	2	2	3	2	
G	G1	2015/winter	C.P.	2	2	5	1	1
H	H1	2015/winter	C.P.	2	3	12	3	
	H2	2015/spring						
	H3	2015/fall						
I	I1	2015/winter	HU	3	1	3	1	1
J	J1	2015/spring	HU	2	2	3	1	1
K	K1	2015/spring	HU	2	3	2	1	1
L	L1, L2, L3	2015/summer	F	1	1	10	3	
M	M1	2015/summer	C.P.	2	2	2	1	1
N	N1	2015/summer	C.P.	3	3	7	1	1
O	O1	2015/summer	N.A.	2	1	3	1	
P	P1	2015/fall	C.P.	3	2	3	1	
Q	Q1, Q2	2015/fall	C.P.	3	1	7	1	

^a^C.P. = Central Plateau, N.A. = Northern Alps, J. = Jura, HU = Hungary, F = France.

Of the total of 109 puppies, 58 were male and 50 female, and for one puppy the sex was not recorded. When the puppies were assessed for their allergy status at three years of age 48 animals classified as allergic (44.0%) and 44 as non-allergic (40.4%); 17 animals (15.6%) could not be followed-up because they were living in new households too far away or the new owners did not consent to participate.

### Bacterial microbiota

In total, for all sequenced samples including the controls 32.6 million read pairs were processed. The primers were detected in 27.3 million read pairs of which 91.6% were merged, while 57.7% of the merged reads passed the quality filtering.

Clustering resulted in 11,219 OTUs and 75.0% of all merged reads mapped to the OTU centroids. The average sequence count per sample was 77,737 ± 5,456 SD. After filtering and rarefaction to a depth of 16,000, three samples as well as the negative and extraction controls were excluded and the OTU table contained 235 samples and 2,119 OTUs.

### Analysis of the bacterial microbiota

**Figure 1:**
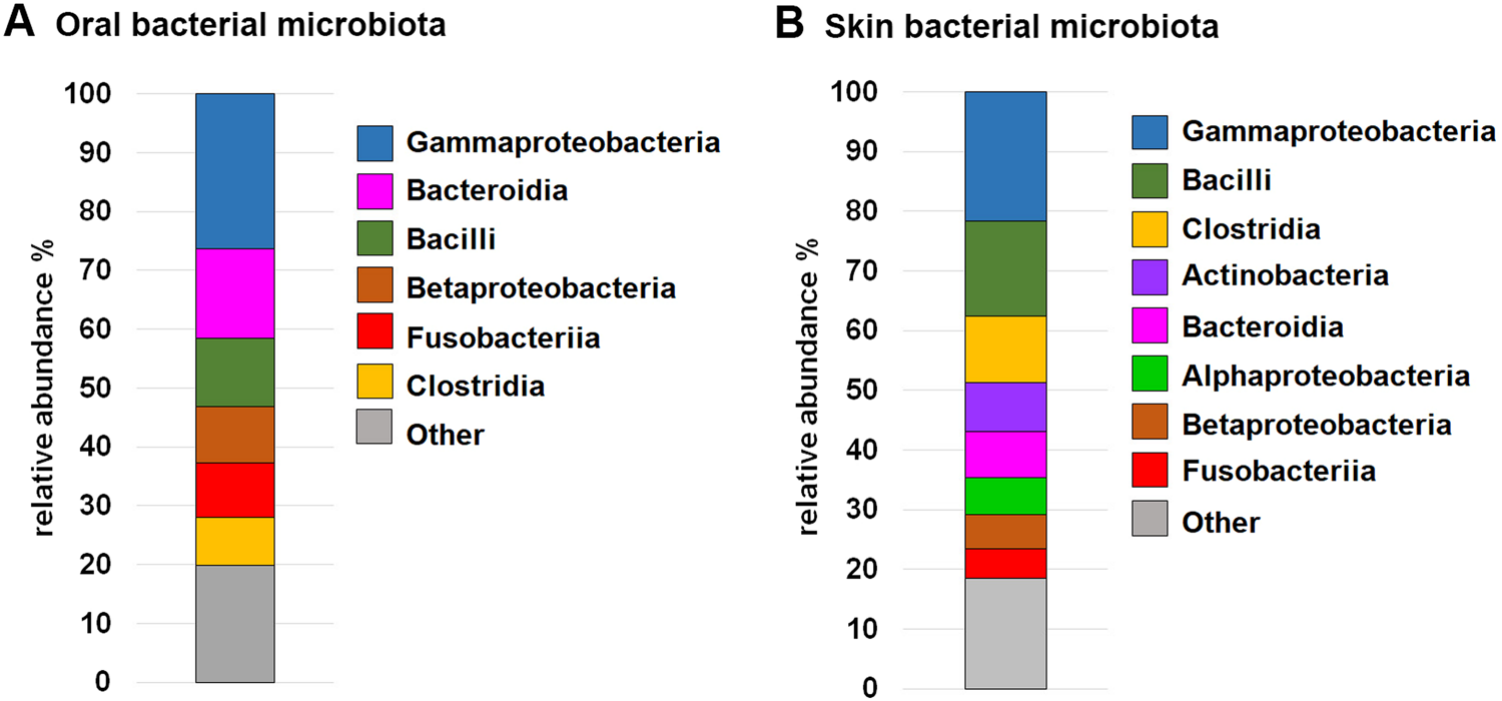
The stacked bar charts show the predominant taxa at class level accounting for ≥ 80% of the relative abundance of the oral (**A**) and skin (**B**) bacterial microbiota.

The oral bacterial microbiota was dominated (>80% of the relative abundance) by the following classes: Gammaproteobacteria (26.3%) and Betaproteobacteria (9.5%) (phylum Proteobacteria, overall relative abundance 40.3%); Bacilli (11.6%) and Clostridia (8.1%) (phylum Firmicutes, overall relative abundance 20.5%); Bacteroidia (15.2%) (phylum Bacteroidetes, overall relative abundance 20.5%); Fusobacteriia (9.3%) (phylum Fusobacteria, overall relative abundance 9.4%) (Fig. 1A).

The predominant classes identified in the cutaneous bacterial microbiota accounted for > 80% of the relative abundance: Gammaproteobacteria (21.7%), Alphaproteobacteria (6.2%) and Betaproteobacteria (5.6%) (phylum Proteobacteria, overall relative abundance 34.6%); Bacilli (15.8%) and Clostridia (11.1%) (phylum Firmicutes, overall relative abundance 29.9%); Actinobacteria (8.1%) (phylum Actinobacteria, overall relative abundance 9.7%); Bacteroidia (7.8%) (phylum Bacteroidetes, overall relative abundance 12.4%); Fusobacteriia (4.9%) (phylum Fusobacteria, overall relative abundance 5.0%) (Fig. 1B).

The α-diversity (microbial diversity of one sample) and the β-diversity (similarity between groups) were assessed for the two sampling sites, oral mucosa and groin. The cutaneous and oral microbiota of the puppies showed statistically significant differences in α-diversity (number of observed OTUs, Shannon diversity index and Chao1 index, *p* < 0.0001) (Supplementary Fig. S1). The groups were also significantly different based on β-diversity (PERMANOVA *F* = 31.34, *p* = 0.0001) as illustrated by principal component analysis (PCA) (Supplementary Fig. S2). Likewise, the cutaneous and oral microbiota of the adult dogs differed significantly in α-diversity (number of observed OTUs, Chao1 and Shannon diversity index, *p* = 0.0001) (Supplementary Fig. S1). The groups differed also based on β-diversity (PERMANOVA *p* = 0.0001, *F* = 16.27) as illustrated by PCA (Supplementary Fig. S2).

**Figure 2:**
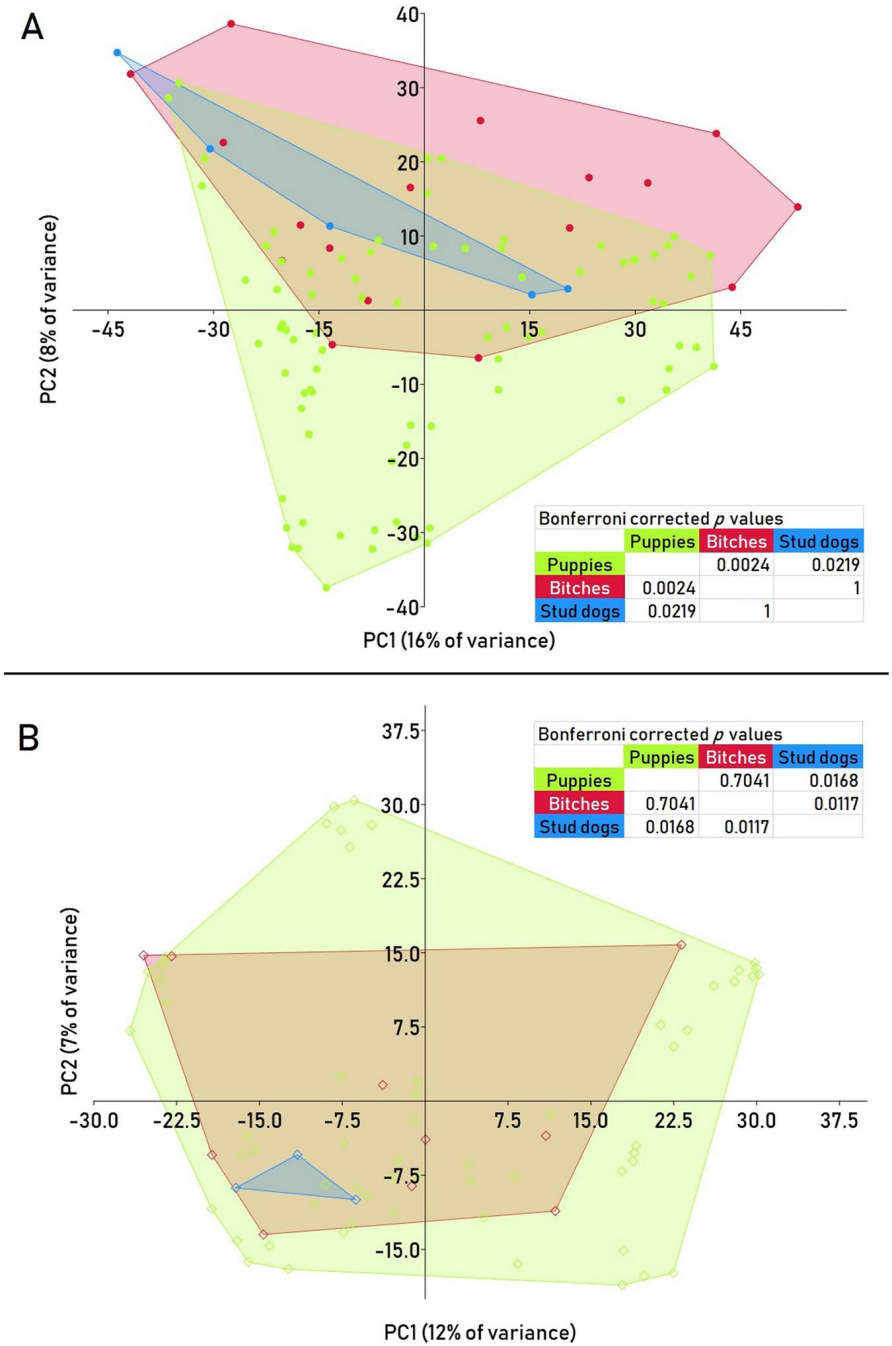
Principal component analysis (PCA) of the bacterial (**A**) and fungal (**B**) skin microbiota of puppies, bitches and stud dogs. shows statistically significant differences between all groups (PERMANOVA bacterial microbiota: *p* = 0.0004, *F* = 2.44; PERMANOVA fungal microbiota: *p* = 0.0078, *F* = 1.53). Bonferroni-corrected *p* values for pairs of groups are shown.

Regardless of age, the canine bacterial skin microbiota showed similar species richness based on the number of observed OTUs (*p* = 0.0654) and Chao-1 index (*p* = 0.0581); only the Shannon diversity index, that considers species richness and evenness, differed significantly (*p* = 0.0368). When comparing puppies to bitches and stud dogs separately, significant differences in α-diversity were observed between puppies and bitches (number of observed OTUs, *p* = 0.0367; Chao1, *p* = 0.0245, Shannon diversity index, *p* = 0.0313). We found significant differences in β-diversity between puppies and adult dogs (PERMANOVA *p* = 0.0001, *F* = 3.91) as well as among puppies, bitches and stud dogs, (PERMANOVA *p* = 0.0004, *F* = 2.44). Here, two pair-wise comparisons were significant: between puppies and bitches (Bonferroni-corrected *p* = 0.0024) and puppies and stud dogs (Bonferroni-corrected *p* = 0.0219) as illustrated by PCA (Fig. 2A). The three most abundant bacterial genera found on the puppies’ skin were *Streptococcus* (9.4%), *Lachnospiraceae* family unclassified genus (4.5%) and *Acinetobacter* (3.7%). The most abundant bacterial genera found on the adult dogs’ skin were *Acinetobacter* (5.3%), *Porphyromonas* (5.0%) and *Streptococcus* (4.1%). Two OTUs identified as *Lachnospiraceae* unclassified genus were significantly different between puppies and adults [false discovery rate (FDR) *p*-value corrected by the Benjamini-Hochberg procedure for multiple comparisons, *p* = 0.002 and *p* = 0.03] with puppies showing a higher mean abundance of counts (346 and 112) when compared to the adult dogs (113 and 26). *Staphylococcus* followed as seventh and fifth most abundant genus in puppies (3.2%) and adult dogs (3.2%), respectively. The mean abundance of counts for the OTU classified as *Staphylococcus* was higher in the bitches (571) than in the puppies (377) and stud dogs (7) (FDR, *p* = 0.049).

The sex of the puppies did not impact the cutaneous bacterial microbiota in terms of α-diversity (number of observed OTUs, *p* = 0.9539; Chao1, *p* = 0.9424, Shannon diversity index, *p* = 0.5995) and β-diversity (PERMANOVA *p* = 0.8922, *F* = 0.67).

Differences between the cutaneous bacterial microbiota of the puppies of the different households were, moreover, analysed for the geographical origin. For this purpose, the Swiss households were assigned to bioregions as defined by the Swiss Federal Office for the Environment (Table 1, Supplementary Fig. S3). Differences between the geographical regions were observed in terms of α-diversity (PERMANOVA *p* < 0.0001, *F* = 3.41). All pairs of geographical regions differed significantly when compared separately, except France and Hungary (Supplementary Fig. S4). No statistically significant differences in α-diversity (number of observed OTUs, Chao1 and Shannon diversity index) were detected between any geographical regions. In addition, the skin microbiota of the puppies differed between the various breeders, with statistically significant differences between all breeders (PERMANOVA *p* = 0.0001, *F* = 4.59) and between nine pairs of breeders (Bonferroni-corrected *p* values for household pairs A and C, *p* = 0.0480; A and E, *p* = 0.0240; A and G, *p* = 0.0360; A and H, *p* = 0.0120; A and Q, *p* = 0.0360; E and L, *p* = 0.0360; E and Q, *p* = 0.0240; H and L, *p* = 0.0240; H and Q, *p* = 0.0240) (Supplementary Fig. S5). The clustering by household could also be visualized by PCA when analyzing the different geographical origins separately (Supplementary Figs S6–S9). In households A, H, E, L and Q two to four litters were cohabitating. A statistically significant difference in β-diversity was only observed for two litters in household H (Bonferroni-corrected *p* value = 0.025) and the two litters in household Q (Bonferroni-corrected *p* value = 0.032) (Supplementary Fig. S6). Litter H2 and H3 were sampled in different seasons, spring and fall of 2015, respectively, and litters Q1 and Q2 were both sampled in the fall of 2015 (Table 1).

**Figure 3:**
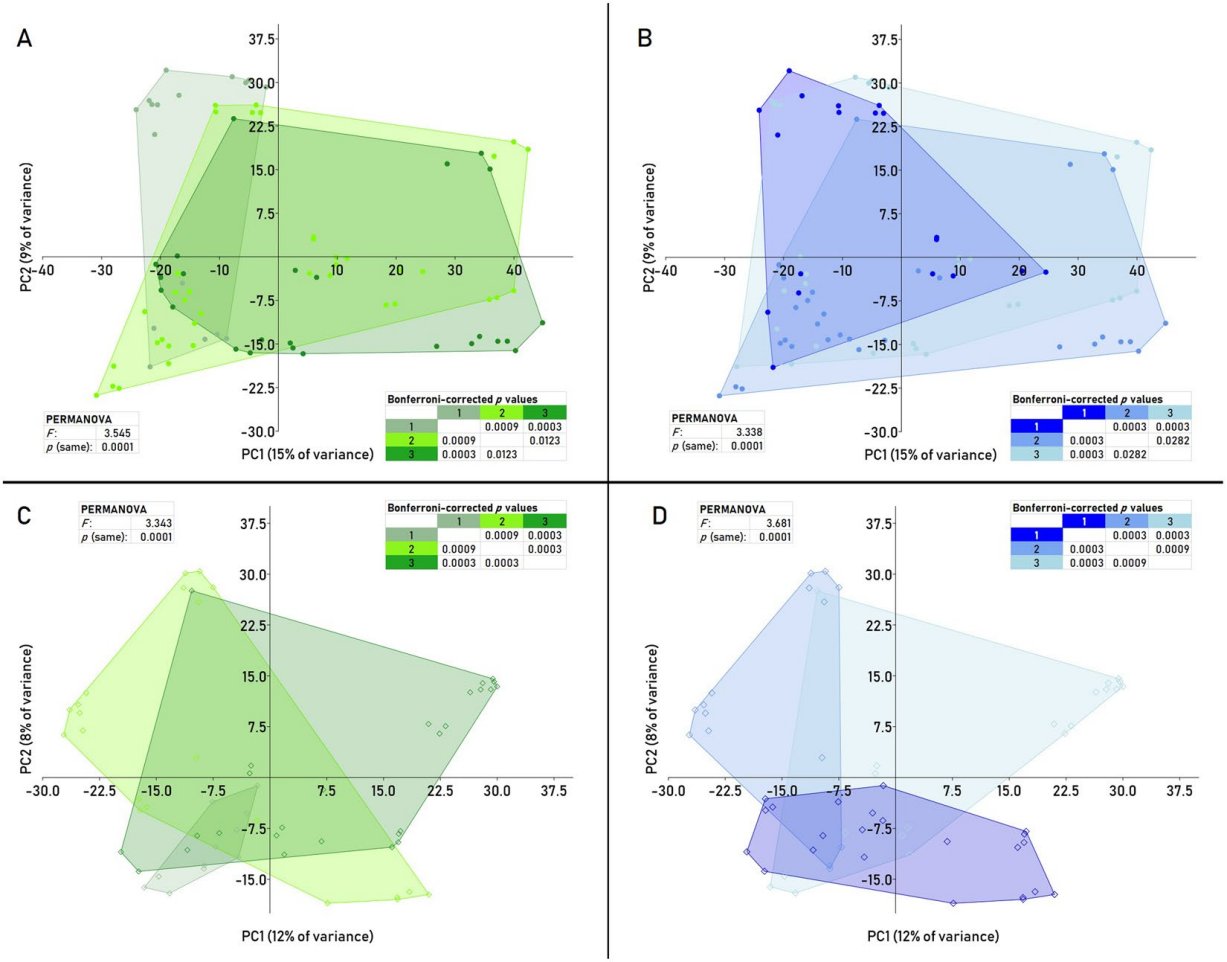
Principal component analysis (PCA) of the bacterial (**A**,**B**) and fungal (**C**,**D**) skin microbiota of puppies comparing the group variables environment (1 = inside, 2 = inside with access to outdoor environment, 3 = outside) (**A**,**C**) and hygiene (1 = low standard, 2 = average standard, 3 = high standard) (**B**,**D**). PERMANOVA and Bonferroni-corrected *p* values are shown.

Differences between the cutaneous bacterial microbiota of the puppies depended on the access to the outdoor environment (Table 1) (PERMANOVA *p* = 0.0001, *F* = 3.55; Bonferroni-corrected *p* values for pairs of groups 1 and 2: *p* = 0.0009; 1 and 3: *p* = 0.0003; 2 and 3: *p* = 0.0123) (Fig. 3A). The level of hygiene of the respective breeders (Table 1) also resulted in significantly different cutaneous bacterial microbiota (PERMANOVA *p* < 0.0001, *F* = 3.34; Bonferroni-corrected *p* values for scores 1 and 2: *p* = 0.0003; scores 1 and 3: *p* = 0.0003; scores 2 and 3: *p* = 0.0282) as illustrated by PCA (Fig. 3B). No significant differences were found between those groups in terms of α-diversity (Supplementary Table S1).

The season of sampling also affected the distribution of the microbial profiles and led to changes in α-diversity between the seasons spring/fall (number of observed OTUs, *p* = 0.0130; Chao1, *p* = 0.0496, Shannon diversity index, *p* = 0.0004), summer/fall (number of observed OTUs, *p* = 0.0282; Chao1, *p* = 0.0081, Shannon diversity index, *p* = 0.0125), winter/fall (number of observed OTUs, *p* = 0.0458; Chao1, *p* = 0.0089, Shannon diversity index, *p* = 0.0514) and to a lesser extent between the seasons spring/summer (number of observed OTUs, *p* = 1.0; Chao1, *p* = 1.0, Shannon diversity index, *p* = 0.0434). Differences were also observed in terms of β-diversity among all groups (PERMANOVA *p* = 0.0001, *F* = 3.54) and between pairs of groups (Bonferroni-corrected *p* values for spring-summer, *p* = 0.0030; spring-winter, *p* = 0.0474; spring-fall, *p* = 0.0030; summer-fall, *p* = 0.0012; summer-winter, *p* = 0.0036; fall-winter, *p* = 0.0030) as illustrated by PCA (Supplementary Fig. S10).

There was no significant difference between the early microbiota of puppies that were classified as allergic and those classified as not allergic after three years, neither based on α-diversity estimators (number of observed OTUs, *p* = 0.9143; Chao1, *p* = 0.8809, Shannon diversity index, *p* = 0.6694) nor in β-diversity (PERMANOVA *p* = 0.3896, *F* = 1.01). Group significance analysis did not reveal significantly different abundance of any of the six OTUs classified as *Staphylococcus* (FDR *p* values ranged from 0.97 to 0.98).

The significance of *p* values obtained for PERMANOVA using unweighted and weighted Unifrac distance did not differ from the above reported results for PERMANOVA using Bray-Curtis distance (Supplementary Table S3).

### Mycobiota

In total, 10.5 million read pairs were processed. The primers were found in 8.7 million read pairs. After primer removal, 91.1% of the read pairs merged and 76.6% of these passed quality filtering. The fungal ITS2 region was extracted from 92.9% of all merged reads. Of all extracted ITS2 regions, 93.5% mapped to the OTU centroids. This resulted in an OTU table containing 106 samples, 5,965 OTUs, and 6.9 million sequences. The average sequence count per sample was 65,282 ± 34 871 SD. After filtering and rarefaction to a depth of 15,000, the negative and extraction controls were excluded and the OTU table contained 103 samples and 2,192 OTUs.

### Analysis of the mycobiota

**Figure 4:**
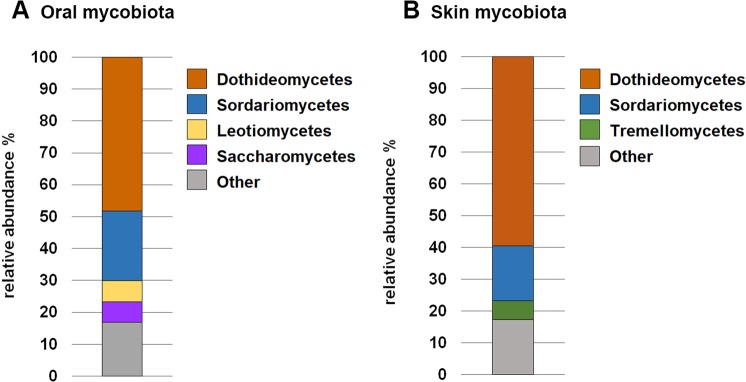
The stacked bar charts show the predominant taxa at class level accounting for ≥ 80% of the relative abundance of the cutaneous (**A**) and the oral (**B**) mycobiota.

The oral mycobiota was dominated by classes belonging to the phylum Ascomycota (overall relative abundance 88.3%): Dothideomycetes (48.2%), Sordariomycetes (21.9%), Leotiomycetes (6.7%) and Saccharomycetes (6.3%) (Fig. 4A). The predominant classes identified in the cutaneous mycobiota that accounted for ≥ 80% of the relative abundance were: Dothideomycetes (59.4%) and Sordariomycetes (17.3%) (phylum Ascomycota, overall relative abundance 88.3%); Tremellomycetes (6.0%) (phylum Basidiomycota, overall relative abundance 10.0%) (Fig. 4B).

Like for the bacterial microbiota, the α-diversity and the β-diversity were assessed for the two sampling sites, oral mucosa and groin. The cutaneous and oral mycobiota of the puppies showed differences in species richness that were significant regarding the number of observed OTUs (*p* = 0.0003) and Chao1 index (*p* = 0.0001), but not based on the Shannon diversity index (*p* = 0.7373). Differences between the samples from different sites were ordinated by PCA (Supplementary Fig. S11A); the sites differed significantly (PERMANOVA *p* = 0.0059, *F* = 1.96). The cutaneous and oral mycobiota of the adult dogs did not display significant differences in α-diversity based on the three estimators number of observed OTUs (*p* = 0.2049), Chao1 (*p* = 0.1579) and Shannon diversity index (*p* = 0.9135) or in β-diversity (PERMANOVA *p* = 0.5504, *F* = 0.95) (Supplementary Fig. S11B).

The canine cutaneous mycobiota showed a similar α-diversity regardless of age when assessing the estimators number of observed OTUs (*p* = 0.7753), Chao1 (*p* = 0.7809) and Shannon diversity index (*p* = 0.1556). When comparing the mycobiota of puppies, bitches and stud dogs separately, significant differences were observed between puppies and stud dogs in the number of observed OTUs (*p* = 0.0300) and Shannon diversity index (*p* = 0.0380), but not for Chao1 (*p* = 0.0911), and between bitches and stud dogs in the number of observed OTUs, *p* = 0.0424 and Shannon diversity index, *p* = 0.0427), but likewise not for Chao1 (*p* = 0.0675). We found significant differences in β-diversity between puppies, bitches and stud dogs in multiple comparisons between all groups (PERMANOVA *p* = 0.0078, *F* = 1.53) and between the pairs of groups puppies/stud dogs (Bonferroni-corrected *p* = 0.0168) and bitches/stud dogs (Bonferroni-corrected *p* = 0.0117) as illustrated by PCA (Fig. 2B).

The three most abundant fungal genera on the puppies’ skin were *Cladosporium* (7.9%), followed by *Didymellaceae* family unclassified genus (3.2%) and *Saccharomyces* (1.7%). *Cladosporium* and *Didymellaceae* family unclassified genus were also the most abundant genera of the adult dogs’ skin (9.2 and 8.4%, respectively) followed by *Didymosphaeriaceae* family unclassified genus (5.1%). No OTU classified as *Didymosphaericaea* or a genus within this family was found in statistically significant different abundance when comparing the puppies and adult dogs. In terms of relative abundance, the genus *Malassezia* ranked 16th in puppies (0.5%) and 29th in adult dogs (0.2%). None of the nine OTU identified as genus *Malassezia* was significantly different in abundance between puppies and adults (FDR *p* values ranged from 0.21 to 0.95).

The sex of the puppies did not impact the cutaneous mycobiota regarding species richness (number of observed OTUs, *p* = 0.3677; Chao1, *p* = 0.5263), but differed in species richness and evenness as estimated by Shannon diversity index (*p* = 0.0326). No significant difference in β-diversity was found between male and female puppies based on PERMANOVA (*p* = 0.7704, *F* = 0.81).

Differences between the cutaneous mycobiota of the puppies from different households was moreover analysed based on geographical origin. The classification of the Swiss households according to bioregion was used like for the bacterial microbiota (Table 1, Supplementary Fig. S3). Significant differences in α-diversity were observed between the geographical regions (PERMANOVA *p* < 0.0001, *F* = 2.69). All pairs of geographical regions differed significantly when compared separately, except Hungary that did not differ significantly from any other region and France that did not differ significantly from the bioregions Northern Alps, Jura and Hungary (Supplementary Fig. S12). In terms of β-diversity, no statistically significant differences were found between any geographical regions.

A clustering of the fungal skin profiles based on the respective household was observed by PCA with statistically significant differences confirmed between all households (PERMANOVA *p* = 0.0001, *F* = 4.7) and between nine pairs of breeders (Bonferroni-corrected *p* values for pairs of breeders A and C, *p* = 0.0198; A and E, *p* = 0.0066; A and H, *p* = 0.0066; A and N, *p* = 0.0330; A and Q, *p* = 0.0132; C and H, *p* = 0.0462; E and H, *p* = 0.0198; H and N, *p* = 0.0396; H and Q, *p* = 0.0264) (Supplementary Fig. S13). The clustering by household could also be visualized by PCA when analyzing the different geographical origins separately (Supplementary Figs S14–S16). In households A, H, E, L and Q two to four litters were cohabitating. Differences in β-diversity between different litters from the same household were obtained by PERMANOVA for households A (*p* = 0.0002, *F* = 4.13) and H (*p* = 0.0092, *F* = 6.45), but not for litters in household Q (Supplementary Fig. S14). Litters A1-A4, H2 and H3 were sampled in different seasons and years, while the two litters from household Q were sampled in the same season and year (Table 1).

Differences in the cutaneous mycobiota of the puppies by duration of daily access to the outdoor environment could be perceived by PCA (Fig. 3C) and proved significant by PERMANOVA between all groups (PERMANOVA *p* = 0.0001, *F* = 3.34) and between pairs of groups (Bonferroni-corrected *p* values for scores 1 and 2: *p* = 0.0009; scores 1 and 3: *p* = 0.0003; scores 2 and 3: *p* = 0.0003). The same was depicted by PCA for groups based on the level of hygiene of the respective households (Fig. 3D), and was proven to be significant by multiple comparison between all groups (PERMANOVA *p* = 0.0001, *F* = 3.68) and between pairs of groups (Bonferroni-corrected *p* values for scores 1 and 2: *p* = 0.0003; scores 1 and 3: *p* = 0.0009; scores 2 and 3: *p* = 0.0009). No significant differences were found between those groups in terms of α-diversity, except for groups with hygiene status 1 and 2 that showed a significant difference based on Shannon diversity index (*p* = 0.0064) (Supplementary Table S2).

The season of sampling had a noteworthy impact on the distribution of the fungal profiles (Fig. 4B) as significant differences in β-diversity were found between all the seasons (PERMANOVA, *p* = 0.0001, *F* = 3.22) and between pairs of seasons (Bonferroni-corrected *p* values for spring-summer: *p* = 0.0006; spring-winter: *p* = 0.0006; spring-fall: *p* = 0.0528; summer-fall: *p* = 0.0378; summer-winter: *p* = 0.0006; fall-winter: *p* = 0.0366) (Supplementary Fig. S17). However, we did not find significant differences in α-diversity between the seasons of sampling (Supplementary Table S4).

Like for the bacterial cutaneous profiles, there was no significant difference in β-diversity (PERMANOVA *p* = 0.0513, *F* = 1.32) between the early microbiota of puppies that were classified as allergic and those classified as not allergic after three years. Regarding the α-diversity the estimators for species richness did not differ significantly (number of observed OTUs, *p* = 0.4010; Chao1, *p* = 0.4675); however, species richness and evenness estimated by Shannon diversity index differed significantly (*p* = 0.03434). Group significance analysis did not reveal significantly different abundance of any of the nine OTUs classified as *Malassezia* (FDR *p* values ranged from 0.74 to 0.99).

Positive correlations between the number of observed of OTUs (*r* = 0.62; p < 0.0001), the Chao1 indices (*r* = 0.55; p < 0.0001) and Shannon diversity indices (*r* = 0.40; p < 0.01), were observed for bacterial and fungal profiles of the puppies’ skin (Supplementary Fig. S18).

There was no significant association between the hygiene status [χ^2^(2) = 3.74, *p* = 0.15], access to the outdoor environment [χ^2^(2) = 1.62, *p* = 0.45] or geographical/bioregion [χ^2^(4) = 5.51, *p* = 0.24] and whether or not the puppies would develop allergy later on.

## Discussion

### The canine oral and skin microbiota

The oral samples served as a control group in this study. The statistically significant differences between the oral and cutaneous bacterial microbiota corroborate the sampling scheme as well as the sequencing and data processing methods, since significant differences between mucosal surfaces and haired skin are to be expected with the mucosal surfaces showing lower diversity^26^. The oral and cutaneous mycobiota were described to vary to a lesser extent^27^, which could explain that we did not find significant differences between the sampling sites in the adult dogs.

The oral microbiota was dominated by Proteobacteria, Firmicutes and Bacteroidia which was observed in a previous study, although with Firmicutes being the most abundant class followed by Bacteroidia and Proteobacteria^28^. The predominant bacterial phyla of the cutaneous microbiota, Proteobacteria, Firmicutes, Actinobacteria, Bacteroidetes and Fusobacteria are in line with previous findings in healthy dogs^26,29,30^. Among the most abundant genera observed in our study were *Acinetobacter*, *Streptococcus* and *Porphyromonas*. Previous studies also found *Streptococcus*^19^ and *Porphyromonas*^19,31^ among the dominant genera of the bacterial cutaneous microbiota in healthy dogs. Interestingly, a recent study of the bacterial skin microbiota of children and their mothers, noted that the genus *Streptococcus* was more abundant in the early-life microbiota^32^, like in our study. In earlier studies using culture-based methods, *Acinetobacter* was among the most common commensal bacteria isolated from the skin of healthy dogs and in was present in at least 50% of healthy dogs in a study using amplicon sequencing^26^. In a recent publication, *Cutibacterium* (formerly known as *Propionibacterium*), an abundant component of the human skin^33^, was found to dominate the canine skin^31^. This genus is likely to be underestimated when amplifying the V4 region only^34^. In this study, targeting regions V3 and V4, we did not find *Propionibacterium* to be predominant on the skin of the groin. A similar observation was made in a previous study targeting V1-V3^26^ that described *Cutibacterium* as predominant in axilla, but not in groin samples. On the human skin, *Cutibacterium* is moreover positively correlated with age and thus less abundant in the early-life microbiota^32^. It would be interesting to assess the development of the canine skin in a longitudinal study to corroborate such differences.

Differences encountered between studies may furthermore be due to different sampling and DNA extraction techniques. For instance, Torres and collaborators^31^ shaved the sample area before swabbing, unlike in our study and the before mentioned ones^19,26,29,35^. Other factors that may influence the results are the DNA extraction technique, as well as the sequencing technology. Apart from methodological factors that may influence the study outcome, characteristics of sample population such as number and breed of dog or the geographical origin are noteworthy. For instance, Cusco and collaborators^30^ observed that the breed explained 9% of the distances between the bacterial skin microbiota of healthy dogs. This confounding factor is not applicable to our study that included only dogs of the same breed.

The oral mycobiota was solely dominated by fungi of the phylum Ascomycota, whereas the skin mycobiota was mainly composed of fungi of the phyla Ascomycota and Basidiomycota. This observation is in line with the previous finding of reduced fungal diversity on mucosal surfaces^27^ and the predominance of Ascomycota and Basidiomycota on the healthy canine skin. Moreover, like in our study, the genus *Cladosporium* was one of the predominant genera on healthy canine skin^27^.

### Differences between the early-life and adult canine skin microbiota

To our knowledge this is the first study to compare the early-life and the adult canine skin microbiota. The adult dogs presented a higher bacterial diversity when compared to the puppies. Differences between bitches and puppies and stud dogs and puppies were observed for the cutaneous bacterial microbiota, but not between bitches and stud dogs. For the human skin it was recently described that differences between mothers and their children may be due to the immune maturation which is linked to hormonal changes^32^. This may furthermore explain differences driven by the sex of the dogs as described in an earlier study in older dogs^29^. We did not observe differences due to the sex of the puppies and we believe that differences between male and female are more likely to become apparent when the animals undergo hormonal changes. Our finding might indicate that the individual microbiota is still under development at this early age, which could explain the differences between puppies and adult dogs.

In contrast, the mycobiota showed no variation between the puppies and the adult dogs. Differences were only observed between puppies and stud dogs and bitches and stud dogs. This leads to the assumption that the bacterial microbiota may be more specific to individuals while the mycobiota is rather shaped by environmental factors. It was previously observed that bacteria are more intrinsic to the individual microbiota than fungi^36,37^.

*Lachnospiraceae* is a family of strict anaerobic bacteria that are frequently found in gut of mammals. They were identified in a previous study as differentially abundant in the perianal region compared to other skin sites^29^. A study on the development of the gut microbiota in German Shepherd dogs found this family to increase in abundance from puppyhood to adulthood^38^, which is in contrast to the increased number of *Lachnospiraceae* on the puppies’ skin in our study. Interestingly, a study on the human microbiota in relation to the postnatal exposure to household disinfectants, found that with the more frequent use of disinfectants, *Lachnospiraceae* were significantly increased in the infant gut microbiota^39^. This observation could explain our finding of increased mean abundance of OTU counts to *Lachnospiraceae* unclassified genus in puppies from environments with higher hygiene scores.

### Factors impacting the early-life canine skin microbiota

Previous studies showed that the microbiota of healthy and allergic dogs differs significantly during disease flares^26^ and treatment^19^. Furthermore, the presence of opportunistic bacteria, such as *S. aureus* in humans^18^ and *S. pseudintermedius* in dogs^40^, and fungi such as *Malassezia* are believed to exacerbate the clinical signs of atopic dermatitis in humans^20^ and dogs^21–25^. Although these staphylococci and this yeast rather cause secondary infections in CAD, we hypothesized that a higher abundance of those in the cutaneous bacterial and fungal microbiota might predispose the individuals for CAD. Less than half of the puppies enrolled in this study were classified as allergic at the age of three years, which corresponds to the generally assumed rate of allergy, indicating that our sampling is representative. We did not observe statistically significant differences in abundance of OTUs belonging to the genera *Staphylococcus* or *Malassezia* between the healthy puppies and those that developed allergy later on. In an earlier study, allergic dogs were shown to have less biodiversity than healthy dogs and to harbor more coagulase-positive staphylococci, such as *S. aureus*^26^. However, it was then unclear, if this was a cause or an effect. Except for the significant difference in the species richness and evenness (estimated by Shannon diversity index) between the fungal microbiota of allergic and non-allergic dogs, we could not find indicators in the early-life microbiota related to the later development of allergy. Our results suggest that the microbial diversity of the early-life microbiota is a poor indicator for the development of CAD.

It was previously hypothesized based on observations from a cross-sectional study on dogs of the same breed in a shared environment that the season of birth affected the skin microbiota^29^. The impact of the season was confirmed in a longitudinal study of 20 households^31^. Although our study does not allow for a temporal comparison due to the cross-sectional study design, we included a comparison based on season of sampling and could show that the season should indeed be considered a possible confounding factor.

The amount of access to the outdoor environment and standard of hygiene both seem to shape the microbiota and are likely to drive the observed significant differences between households. Interestingly, neither of the two served as predictor on whether or not the puppies would become allergic after three years and thus, we could not confirm the observation made in humans, that higher microbial exposure leads to protection from allergy^9,10^. It should be noted though, that our study took place in the first three weeks of life of the puppies while they were still with their mothers and factors influencing the development of allergy could arise later when they were with new owners. Since the variables household, hygiene and environment seem to impact the composition of the microbiota of the skin significantly, we strongly recommend taking these confounding factors into account when conducting microbiome studies in dogs from different households. Five households raised more than one litter and although some clustering was visualized by PCA, the bacterial skin microbiota only differed significantly between two litters from two households and the skin mycobiota only differed significantly between two litters from one household. A temporal factor, such as year and season of sampling could only be attributed to one household (H). Other litters sampled in different years and seasons (A and E) did not display significant differences, so that the underlying reasons for variation remain unclear. However, this observation is in agreement with an earlier study that cohabitation has a significant impact on the dog skin bacterial community^31^. When using a dog model to study AD, experiments would need to ensure that the animals are kept under the same conditions to be able to draw conclusions free from bias. It would moreover be advisable, due to significant individual variation of the skin microbiota, to compare healthy and allergic microbiota by sampling affected versus unaffected areas of the same individual rather than comparing healthy and allergic individuals as previously suggested^30^.

Interestingly, we found a positive correlation between the bacterial and the fungal diversity of the puppies’ skin microbiota. It seems logical to assume that the more diverse bacteria are present in a household/kennel, the more fungi would be present, too. Nevertheless, this is in contrast to previous observations for house-dust that suggested a negative correlation of fungal and bacterial species diversity^41–43^.

### Methodological limitations

A general limitation of amplicon sequencing of the 16S rRNA gene of bacteria as well as the ITS regions of fungi is the small amplicon size that does not exploit all the taxonomic information available. The choice of the region to be amplified impacts the overall coverage of phyla, classes and genera. The primer pair used in our study for amplification of the V3-V4 region of the 16S rRNA gene was previously evaluated by Klindworth and collaborators^44^ who concluded that it presented the best combination of overall coverage and phylum spectrum with the SILVA database^45^, that we used for taxonomic classification in this study. However, as a limitation they described that if one mismatch is accepted, some archaeal sequences are also detected with this primer pair. Another important issue in microbiome studies is the presence of exogenous DNA revealed in negative controls that can arise from sequencer errors (base miscalls resulting in barcode cross talk), or laboratory contaminants (microbial DNA inherent to extraction kits, “kitome”, or other laboratory reagents). This exogenous DNA may have an impact on the data interpretation particularly in low-microbial-biomass samples such as skin. Different types of control samples as well as computational approaches have been proposed in recent years to address this problem^46,47^, but no standard approach is available to date. A recent study concluded that the controversial method of negative-control filtering may lead to erroneous removal of important data especially in low biomass samples such as skin samples^46^. The authors, moreover, described abundance filtering as more accurate. In this study, we used conservative abundance filtering to limit the loss of potentially important data. In our study, carried out between 2014 and 2015, we included only one extraction control and the negative PCR controls for the batchwise library preparation; we would opt for the inclusion of a mock community dilution series as a control as recommended by Karstens and colleagues^46^.

The sampling groups puppies, bitches and stud dogs vary in size due to natural reasons (size of litter per bitch) as well as logistical reasons (not all the stud dogs fathering the litters were available for sampling). This could have influenced the comparisons between those groups.

Our study design only considered a single timepoint of sampling and A follow-up sampling of the three-year-old dogs when they were examined for the allergy status, would have allowed for a temporal assessment of the development of the puppies microbiota and the possible shifts in microbial diversity between allergic and not allergic dogs.

## Conclusion

To our knowledge, this is the largest cohort study of the cutaneous bacterial and fungal microbiota of dogs to date. We showed that not only the age, but also rearing and environmental factors, influenced the development of a specific skin microbiota. However, no association between those factors and the later development of allergy was observed. Even if a standardized sampling protocol is used, the cutaneous microbiota of dogs from different households is likely to vary strongly, which needs to be taken into account in clinical studies. Our results suggest that the individual early-life microbiota does not predispose for the development of CAD in WHWT.

## Methods

### Sampling and data collection

Over a 1.5 year period (May 2014 – November 2015) 288 samples from 29 litters (A1-Q2) of 143 WHWT from 17 different breeders (A-Q) from Switzerland (n = 13), Hungary (n = 3) and France (n = 1) were collected. The Swiss households were in the bioregions Plateau (n = 10), Northern Alps (n = 2) and Jura (n = 1), the Hungarian households were in the Budapest area and the French household was in the Grand Est region in north-eastern France (Supplementary Fig. S3). The map was built using a free online tool including the bioregion information of the Swiss Federal Office for the Environment (https://map.geo.admin.ch, consulted on September 7, 2019). The breeders gave their informed consent to participate in this study and each household was given a score for hygiene depending on the weekly frequency of cleaning the bedding of the puppies and the floor (1 = low, once per week; 2 = average, 2 to 3 times per week; 3 = high, 4–7 times per week). A score was also given for access of the dogs to the outdoor environment (1 = mostly inside, less than 1 hour outside per day; 2 = inside, but with > 1 to 3 hours outside activity per day, 3 = more than 3 hours outside per day). Samples were obtained by rubbing sterile dry nylon fiber tipped applicators (Milian SA, Niederwil, AG, Switzerland) 40 times over a 4 cm^2^ area of the skin of the groin (n = 142) and 40 times over the hard palate to sample the the oral mucosa (n = 143) of 109 puppies during their first three weeks of life and 34 parent dogs (bitches, n = 28; stud dogs, n = 6). The sampling took place covering the four seasons: spring (36 dogs), summer (59 dogs), fall (21 dogs) and winter (28 dogs). The tips of the swabs were transferred in sterile manner into PowerBead tubes (PowerSoil® DNA Isolation Kit, Mobio Laboratories, Carlsbad, CA, USA) containing 750 μl solution. The samples were kept at room temperature, sent to the laboratory overnight and total genomic DNA was extracted upon arrival following the manufacturer’s instructions. The DNA extraction procedure was performed with an extraction control tube containing only reagents.

The allergy status of the puppies enrolled in the study was assessed when the animals were three years old based on the on the current clinical Favrot’s criteria^48^.

The protocols were approved and permission for animal experimentation was granted in accordance with regulations of the Swiss legislation (Federal Animal Protection Law, 455, https://www.admin.ch/opc/ de/classified-compilation/20022103/index.html) by the *Kanton Zürich Gesundheitsdirektion Veterinäramt* ZH199/201/4 (No. 25693). The sampling kits were prepared in a biosafety level 2 laboratory; a separate kit was prepared for every dog and stored at room temperature in a hardcover plastic sleeve until use. All laboratory methods were performed in a biosafety level 2 laboratory in accordance with the Swiss Accreditation and Designation Ordinance (dated June 17, 1996).

### Library preparation and amplicon sequencing

DNA was quantified by fluorometry (Quantifluor, Promega, Madison, WI, USA) and all samples were screened for the presence of the 16S rRNA gene as described by Vidal^49^. To check for the presence of fungal DNA a PCR targeting ITS2 was performed. The ITS2 PCR was carried out with primers ITS3 (5′- GCA TCG ATG AAG AAC GCA GC -3′) and ITS4 (5′- TCC TCC GCT TAT TGA TAT GC -3′)^50^ in a volume of 30 µL with 1X PCR buffer (Solis BioDyne, Tartu, Estonia), 2 mM MgCl_2_, 0.4 µM forward and reverse primer (Microsynth, Balgach, Switzerland), 200 µM dNTPs (Roche Diagnostics GmbH, Mannheim, Germany), 1.5 U thermostable DNA FIREPol® Polymerase (Solis BioDyne) and 2 µL of DNA template. The following cycling conditions were applied: 94 °C for 3 min, followed by 35 cycles of 95 °C for 30 s, 50 °C for 30 s, and 72 °C for 1 min, and a final elongation step at 72 °C for 8 min. Samples that were positive in the screening, were further processed. Two-step amplicon libraries for the 16S rRNA hypervariable V3-V4 region were prepared from 238 samples (mouth, n = 134; skin, n = 104) from 137 dogs (puppies, n = 106; bitches, n = 25; stud dogs, n = 6) representing 28 litters from the 17 breeders. Two-step amplicon libraries for ITS2 were prepared from 103 samples (mouth, n = 31; skin, n = 72) from 78 dogs (puppies, n = 61; bitches, n = 13; stud dogs, n = 4) representing 24 litters from 15 breeders.

To sequence the V3-V4 of the 16S rRNA gene of bacteria PCR libraries were generated with the primer pair 341F^44^ and 806R^51^ including the default Illumina overhang adapter sequences using a high-fidelity polymerase (FastStart™ High Fidelity, Roche Diagnostics, Rotkreuz, Switzerland) in a total volume of 25 µL. The mix contained 1X PCR buffer with 1.8 mM MgCl_2_, 200 µM dNTPs, 0.4 µM of each primer and 1.25 U polymerase and 2 µL of DNA template. The cycling conditions were as follows: 95 °C for 3 min, followed by 15 cycles of 95 °C for 30 s, 50 °C for 30 s, and 72 °C for 1 min, followed by 20 cycles of 95 °C for 30 s, 55 °C for 30 s, and 72 °C for 1 min, and a final elongation step at 72 °C for 8 min. To sequence the ITS2 region of fungi libraries were generated with the primer pair ITS3 and ITS4^50^ including the default Illumina overhang adapter sequences using the high-fidelity polymerase and concentrations as described above in a total volume of 25 µL. The cycling conditions were as follows: 95 °C for 3 min, followed by 35 cycles of 95 °C for 30 s, 50 °C for 30 s, and 72 °C for 1 min, and a final elongation step at 72 °C for 8 min. Subsequently, amplicons were purified using a magnetic bead capture kit (AMPure XP, Beckman Coulter, Brea, CA, USA) and quantified and quality-checked using the Agilent DNA 7500 Kit (Agilent Technologies, Waldbronn, Germany). Negative controls were included in all steps to control for contamination at the library preparation step and were sequenced along with the extraction controls. In a second PCR dual indices and Illumina sequencing adapters were attached using the Nextera XT Index Kit. Subsequently, the amplicons were purified as described above, quantified, and quality-checked using the Fragment Analyzer dsDNA 935 Reagent Kit (Agilent Technologies). Illumina-generated PhiX control libraries and our amplicon libraries were denatured using fresh NaOH and combined. Subsequently, the Illumina MiSeq platform and the MiSeq Reagent kit V3 were used to sequence (2 × 301 nt) the libraries.

### 16S rRNA gene amplicon processing

The produced paired-end reads that passed Illumina’s chastity filter were subjected to demultiplexing and trimming of Illumina adaptor residuals (no further refinement or selection). Processing of the V3-V4 amplicon sequences was performed similarly to^52,53^. The sequence data was first filtered to remove reads containing ambiguous bases, after which the reads were screened for the presence of the V3 or V4 amplicon primer at the start of the read only, allowing one mismatch. After primer removal, the read pairs were merged using USEARCH version 8.0.1623^54,55^. The minimum length of the merged read pairs was set at 380 bases and the maximum length at 438 nt. Sequences were then quality filtered (max. expected error rate 0.002) and clustered into operational taxonomic units (OTUs), in line with the UPARSE pipeline^56^, using the following settings: –uparse_maxdball 1500, only de novo chimera checking, and usearch_global, using all merged reads, with –maxaccepts 8 –maxrejects 64 –maxhits 1. Before clustering, the sorted reads were checked against the Illumina PhiX RTA reference, using both local and global alignment (USEARCH with –id 0.5 –query_cov 0.5 -strand both) to exclude the possibility that PhiX reads were included during clustering. The most abundant sequence of each OTU was selected and assigned a taxonomy using QIIME version 1.8.0^57^, the RDP classifier^58^, with a minimum confidence of 0.8, and the SILVA rRNA database v119^45^. The alignment of the 97% representative 16 S ribosomal DNA (rDNA) sequence set, provided by the QIIME developers, was first trimmed to the V3-V4 region^59^, and this alignment was converted to a set of gap-free nonredundant sequences. This set was used to retrain the RDP classifier.

### ITS amplicon processing

Processing of the ITS amplicons was performed similarly to^53^ with minor changes. The ITS sequence data was first filtered to remove reads shorter than 80 nt and reads containing ambiguous bases, after which the reads were screened for the presence of the ITS primer, allowing one mismatch. The read pairs, starting with the forward or reverse primer and containing it only once, were then merged using USEARCH version 8.0.1623^54,56^ after removal of the primers, allowing “staggered” alignments and a length between 70 and 590 nucleotides. Next, the merged reads pairs were quality filtered (max. expected error rate 0.002). The remaining sequences were checked for the presence of PhiX (usearch_local with –id 0.5 –query_cov 0.5 –strand both) and chimeric sequences (uchime_denovo). Then, the fungal ITS2 region was extracted from all sequences (to which the used ITS primers were reattached) using ITSx version 1.0.11 with fungal models only and otherwise default parameters^60^. All regions extracted from sequences identified as chimeric or not passing the quality filter were removed. Thus, only ITS2 regions (min. length 60 nt) originating from the quality-filtered chimeric-free sequences were clustered, after which all extracted ITS regions were mapped to the OTU centroids (usearch_global with –query_cov 0.90 –target_cov 0.90). The most abundant sequence of each OTU was selected using QIIME version 1.8.0^57^ and assigned a taxonomical classification using the RDP classifier^58^, with a minimum confidence of 0.8, and the ITS2 regions extracted by ITSx from the UNITE database version 7.1 (QIIME release; version version 7.2; file: sh_refs_qiime_ver7_dynamic_s_01.12.2017.fasta)^61^.

### Statistical analysis

The negative and extraction controls were not removed from the dataset to avoid erroneous removal of important data, which is in line with the recent observation that negative-control filter is less accurate than other methods such as the abundance filter^46^. To remove spurious OTUs as recommended by Navas-Molina^62^, a further level of filtering based on OTU abundance was performed to discard OTUs with a number of sequences < 0.001% of the total number of sequences. The sequencing depth was normalized by rarefying the 16S rRNA gene and ITS2 datasets randomly to 16 000 and 15 000 reads per sample, respectively. Paleontological Statistics (PAST version 3.12) software^63^ was used for diversity analyses. To assess the α-diversity (microbial diversity of one group) we used two estimators for the species richness - the number of observed OTUs and Chao1 index - and one estimator for species richness and evenness – the Shannon diversity index. The statistical difference of those estimators between groups was analyzed by two-sample t-test/Wilcoxon Mann–Whitney test (normally/non-normally distributed data). To illustrate difference between groups, data ordination by principal component analysis (PCA) was used on the log2-normalized OTU datasets and differences between microbial profiles were statistically assessed by one-way permutational multivariate analysis of variance (PERMANOVA, 9999 permutations) with Bray-Curtis similarity distance. The *p* values were corrected for multiple comparisons using Bonferroni correction; *p* values < 0.05 were considered statistically significant. For the bacterial data PERMANOVAs (9999 permutations) using weighted and unweighted Unifrac distances were additionally performed in R v.3.6.0^64^ using the packages phyloseq (v.1.28.0)^65^ and vegan (v2.5–5)^66^.

Taxonomic summaries were created using the QIIME script summarize_taxa_through_plots.py.

The script group_significance.py was used to compare OTU frequencies in sample groups. To avoid variance errors and spurious significance for low abundance OTUs, group significance was calculated on OTUs present in at least 25% of the samples. A false discovery rate (FDR) with *p* value corrected by the Benjamini-Hochberg procedure for multiple comparisons was considered significant when < 0.05. Correlation between diversities expressed as Pearson correlation coefficient *r* and chi-square [χ^2^(degrees of freedom)] as well as the corresponding *p* values were calculated using PAST^63^.

### Ethical approval and informed consent

Breeders gave their informed consent to participate in the study. All laboratory methods were performed in a biosafety level 2 laboratory in accordance with the Swiss Accreditation and Designation Ordinance (dated June 17, 1996). The protocols were approved and permission for animal experimentation was granted in accordance with regulations of the Swiss legislation, Federal Animal Protection Law 455 (https://www.zuerchertierschutz.ch/fileadmin/user_upload/Tierschutzthemen/pdf/Tierschutzgesetz_e.pdf) after review by the Committee on Animal Experimentation [*Kantonale Tierversuchskommission* (R-KTVK)] of the Canton of Zurich Department of Health ZH199/201/4, No. 25693.

## Supplementary information

Supplementary Information

## Data Availability

The Next Generation Sequencing data are publicly available at NCBI’s Sequence Read Archive (https://www.ncbi.nlm.nih.gov/sra/) under SRA accession number PRJNA498236.
