# Supplementary material for: Impact of the early-life skin microbiota on the development of canine atopic dermatitis in a high-risk breed birth cohort
Source: Sci Rep. 2020 Jan 23;10:1044. doi: 10.1038/s41598-020-57798-x (PMC6978374; doi:10.1038/s41598-020-57798-x)
Supplement: Supplementary file 1 — Supplementary Information [file 41598_2020_57798_MOESM1_ESM.pdf]

Impact of the early-life skin microbiota on the development of canine atopic dermatitis in a high-risk breed birth cohort

Rodriguez-Campos S.[1,2], Rostaher A.[3], Zwickl L.[3], Fischer N.[3], Brodard I.[1], Vidal S.[1,4], Brandt B.W.[5], Favrot C. [3], Perreten V. [1]*

[1] Institute of Veterinary Bacteriology, Vetsuisse Faculty, University of Bern, Länggassstrasse 122, CH-3012 Bern, Switzerland

[2] Bacteriology and Mycology Unit, Faculty of Veterinary Medicine, Norwegian University of Life Sciences, Ullevålsveien 72, 0454 Oslo, Norway

[3] Clinic for Small Animal Internal Medicine, Vetsuisse Faculty, University of Zurich, Winterthurerstrasse 260, 8057, Zurich, Switzerland

[4] current address : Gnubiotics Sciences SA, Microbiome Research, StartLab / Biopôle, Bat SE-B, Route de la Corniche 5, 1066 Epalinges, Switzerland

[5] Department of Preventive Dentistry, Academic Centre for Dentistry Amsterdam (ACTA), University of Amsterdam and Vrije Universiteit Amsterdam, Gustav Mahlerlaan 3004, 1081 LA, Amsterdam, The Netherlands

*Corresponding author. Mailing address: Institute of Veterinary Bacteriology, University of Bern, Länggassstrasse 122, CH-3012 Bern, Switzerland, Phone: +41 31 631 24 30. Fax: +41 31 631 26 34. E-mail: vincent.perreten@vetsuisse.unibe.ch.

Supplementary Figures

Supplementary Figure S1. Comparison of the α-diversity of the cutaneous and oral bacterial microbiota of the puppies (**A**) and adult dogs (**B**) showing box plots of the observed number of operational taxonomic units (OTUs), Shannon diversity and Chao1 indices including average of the mean (AM) and standard error of the mean (SEM). Statistical significance is indicated (***p < 0.001; ****p < 0.0001).

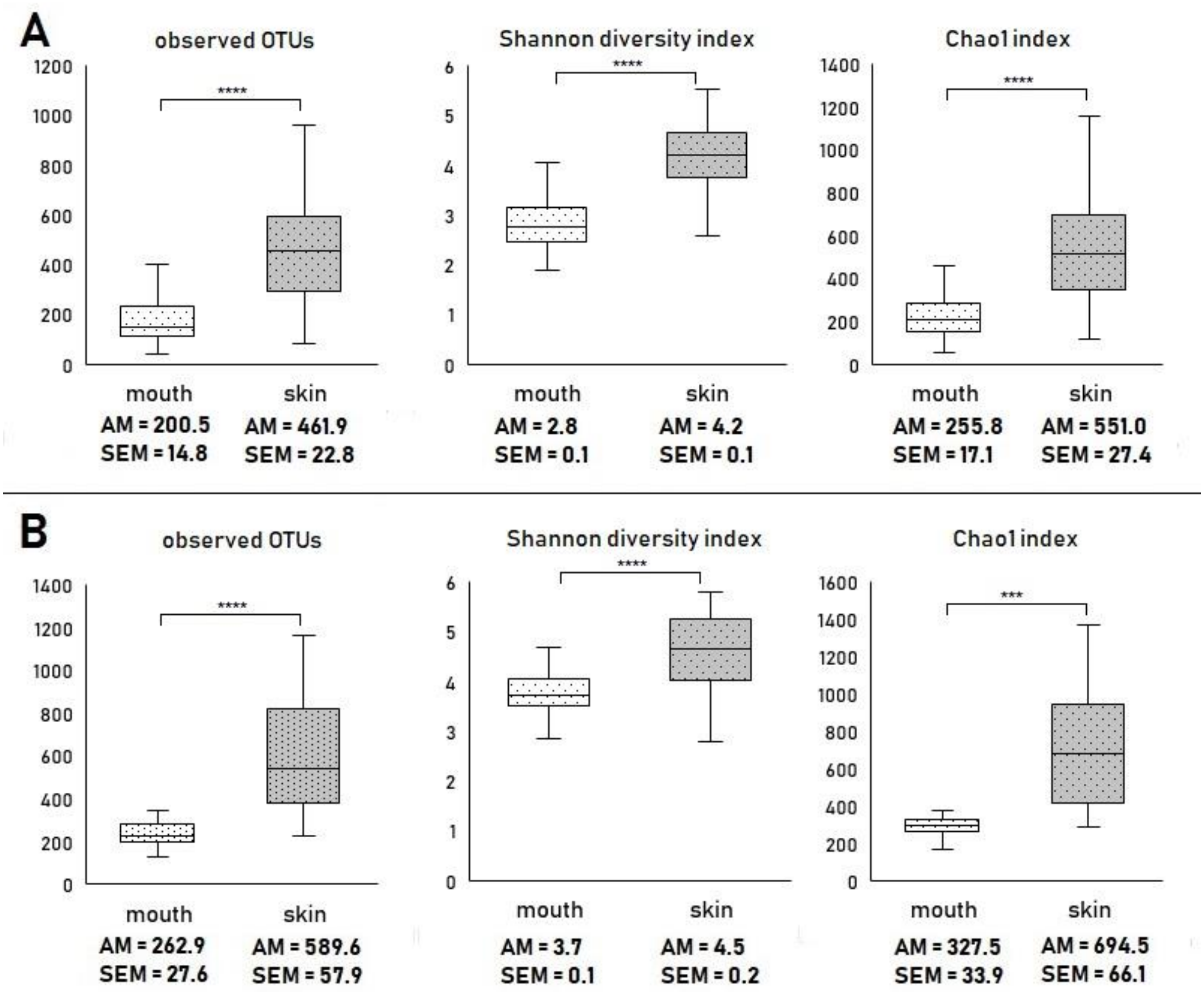

Supplementary Figure S2. Visual comparison by principal component analysis (PCA) of the cutaneous (gray) and oral (black) bacterial microbiota of the puppies (A) and adult dogs (B); PERMANOVA *p* and *F* values are shown.

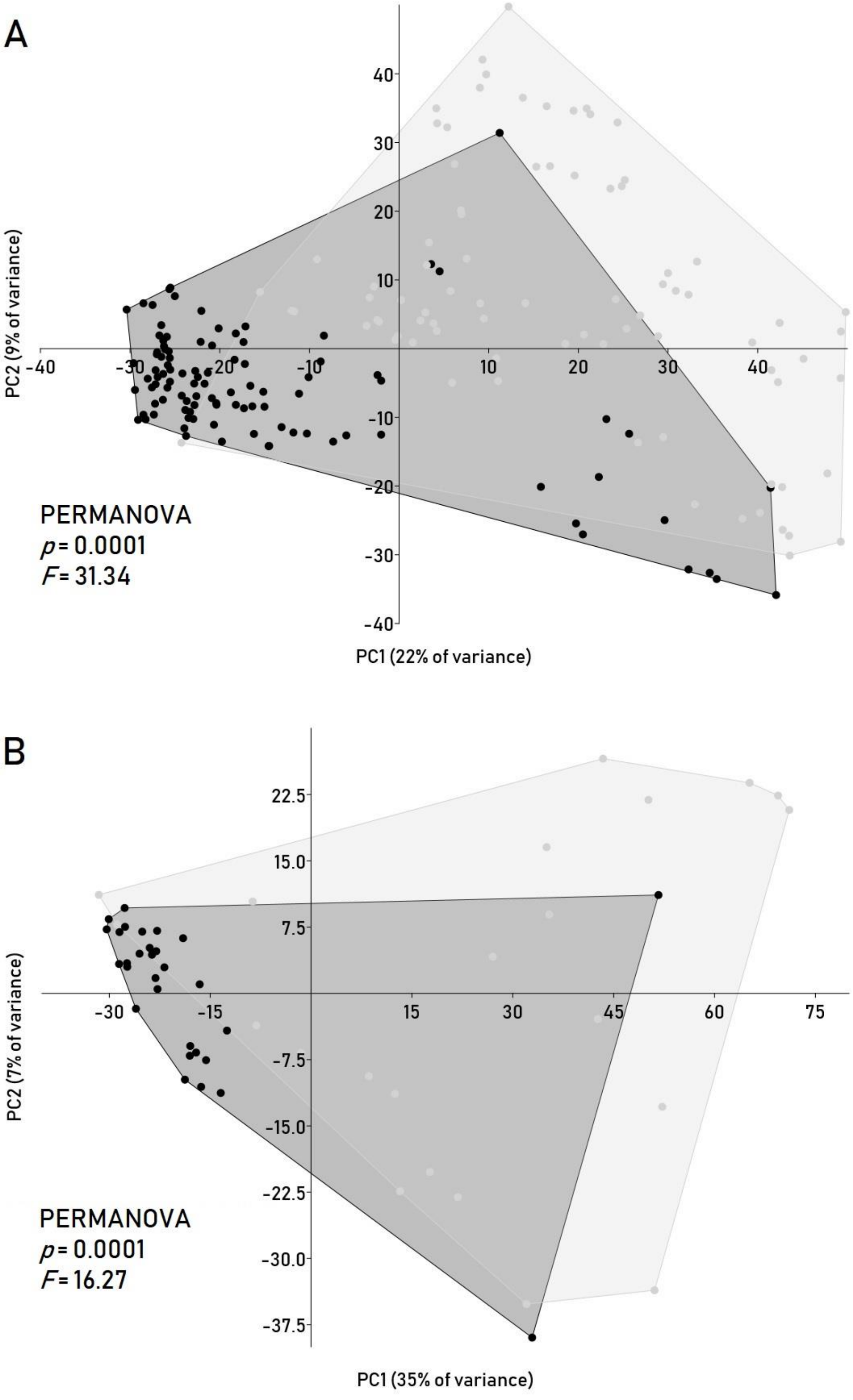

Supplementary Figure S3. Map of Switzerland created with the online tool of the Swiss Federal Office of Topography swisstopo (https://map.geo.admin.ch/, consulted on September 7, 2019) with the following specifications: white background, topic FOEN, include layers for the six biogeographical regions (bioregions) provided by the Swiss Federal Office for the Environment. The locations of the Swiss households (A-H and M-Q) are indicated with a red marker that was set using the tool's built-in Draw & Measure option. The three Hungarian households were all in the Budapest area and the French household was in the Grand Est region in north-eastern France (locations not shown).

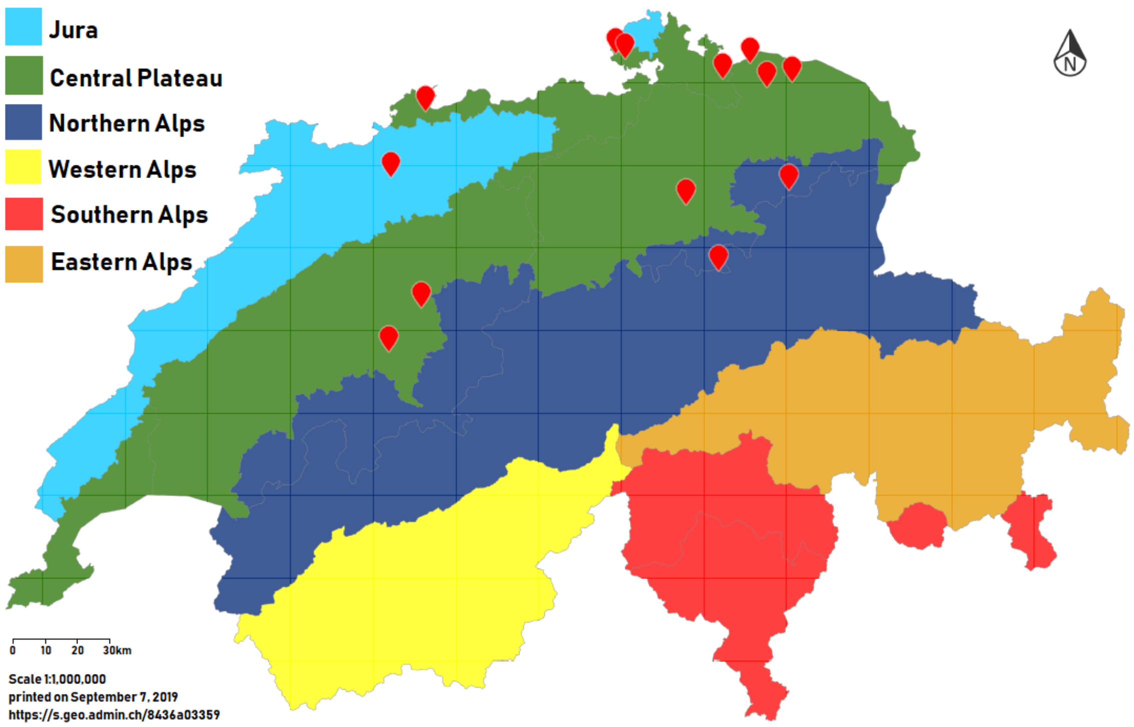

Supplementary Figure S4. Principal component analysis (PCA) of the bacterial skin microbiota of the puppies according to geographical origin. The difference between all groups was significant by PERMANOVA (*p* = 0.0001, *F* = 3.41). Statistically significant Bonferroni corrected *p* values are highlighted in yellow.

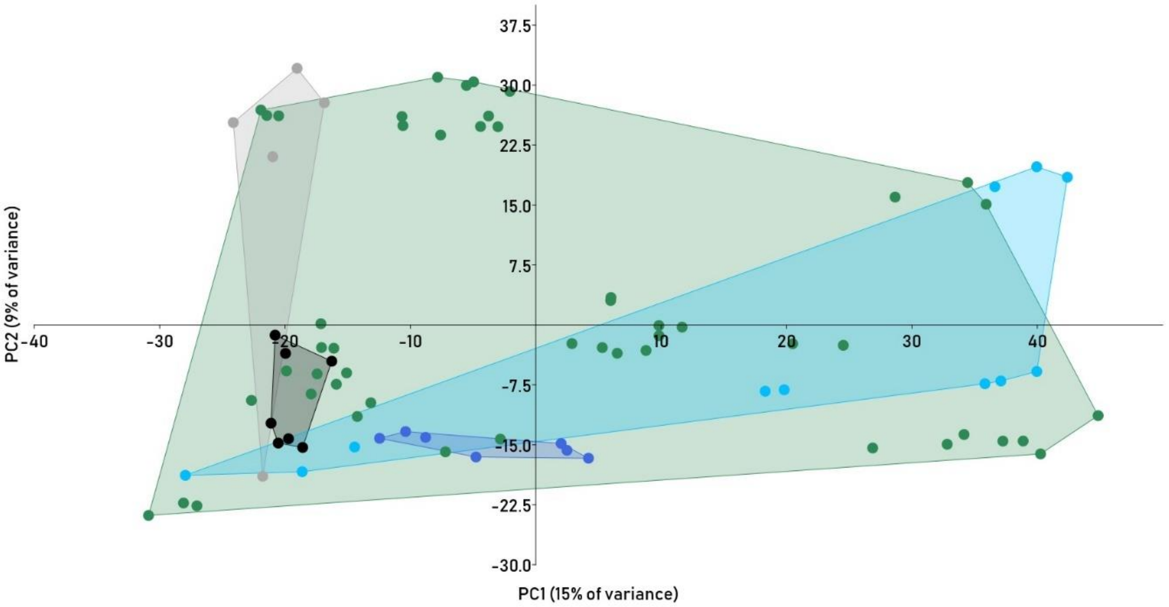

Bonferroni corrected *p* values					
	Central Plateau	France	Hungary	Jura	Northern Alps
Central Plateau		0.008	0.002	0.007	0.016
France	0.008		0.116	0.004	0.01
Hungary	0.002	0.116		0.003	0.01
Jura	0.007	0.004	0.003		0.042
Northern Alps	0.016	0.01	0.01	0.042	

Supplementary Figure S5. Principal component analysis (PCA) of the bacterial skin microbiota of the puppies according to owners. The difference between all groups was significant by PERMANOVA (p = 0.0001, F = 4.59), and between nine pairs of groups as indicated by Bonferroni-corrected p values shown in the table below. Statistically significant p values are highlighted in yellow.

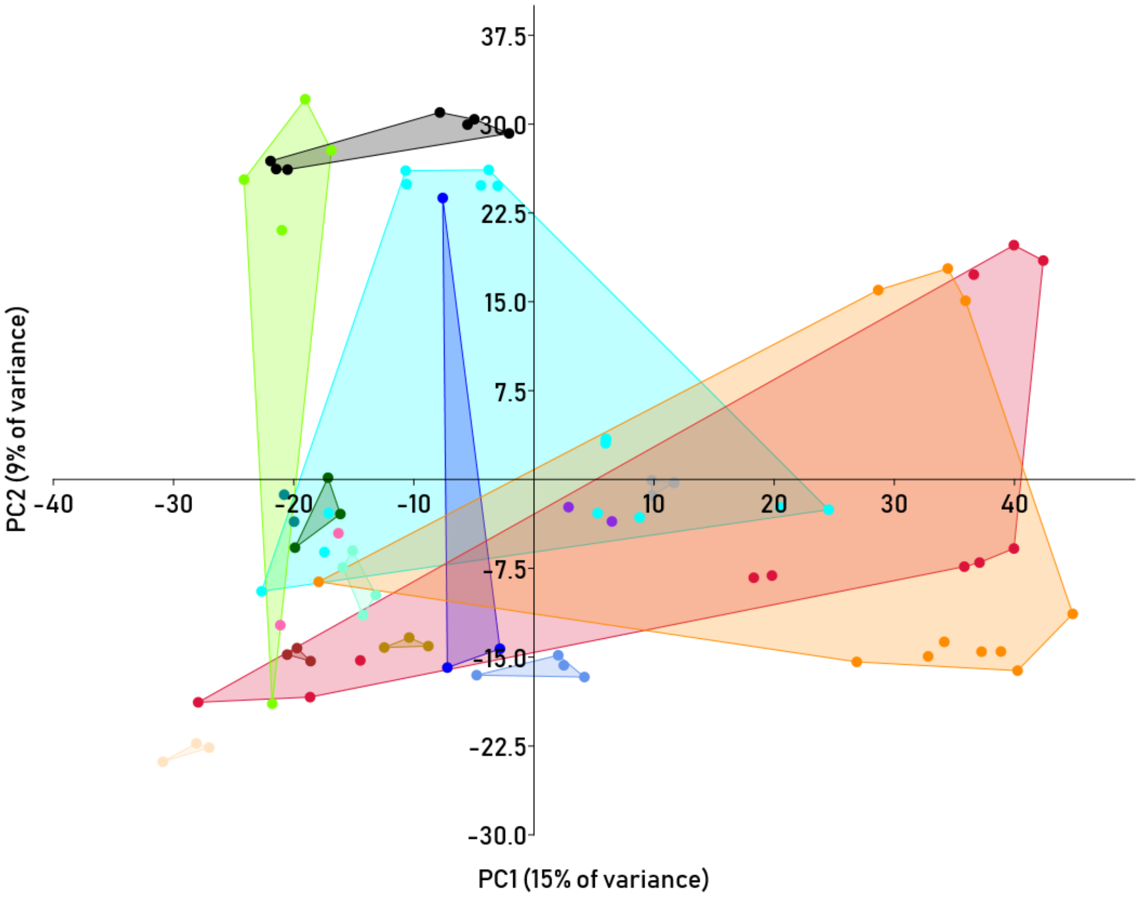

Bonferroni-corrected p values

	A	B	C	D	E	F	G	H	I	J	K	L	N	O	P	Q
A		1	0.048	1	0.024	0.168	0.036	0.012	0.768	0.204	1	0.228	0.384	0.48	1	0.036
B	1		1	1	1	1	1	1	1	1	1	1	1	1	1	1
C	0.048	1		1	1	1	1	0.084	1	1	1	1	1	1	1	0.396
D	1	1	1		1	1	1	1	1	1	1	1	1	1	1	1
E	0.024	1	1	1		0.468	0.072	0.108	1	0.372	1	0.036	0.528	0.816	1	0.024
F	0.168	1	1	1	0.468		1	0.348	1	1	1	1	1	1	1	0.972
G	0.036	1	1	1	0.072	1		0.12	1	1	1	1	1	1	1	0.552
H	0.012	1	0.084	1	0.108	0.348	0.12		1	0.408	1	0.024	0.324	0.396	0.264	0.024
I	0.768	1	1	1	1	1	1	1		1	1	1	1	1	1	1
J	0.204	1	1	1	0.372	1	1	0.408	1		1	1	1	1	1	1
K	1	1	1	1	1	1	1	1	1	1		1	1	1	1	1
L	0.228	1	1	1	0.036	1	1	0.024	1	1	1		1	1	1	0.216
N	0.384	1	1	1	0.528	1	1	0.324	1	1	1	1		1	1	1
O	0.48	1	1	1	0.816	1	1	0.396	1	1	1	1	1		1	0.984
P	1	1	1	1	1	1	1	0.264	1	1	1	1	1	1		0.984
Q	0.036	1	0.396	1	0.024	0.972	0.552	0.024	1	1	1	0.216	1	0.984	0.984	

Supplementary Figure S6. Principal component analysis (PCA) of the bacterial skin microbiota of the puppies belonging to households of the Central Plateau bioregion. The difference between all groups was statistically significant by PERMANOVA (p = 0.0001, F = 5.55). The differences between litters from the same household (A, H and Q) was statistically significant by PERMANOVA (A: p = 0.0001, F = 9.82; H: p = 0.0009, F = 7.12; Q: p = 0.0269, F = 9.97). Statistically significant p values between pairs of households and litters are highlighted in yellow.

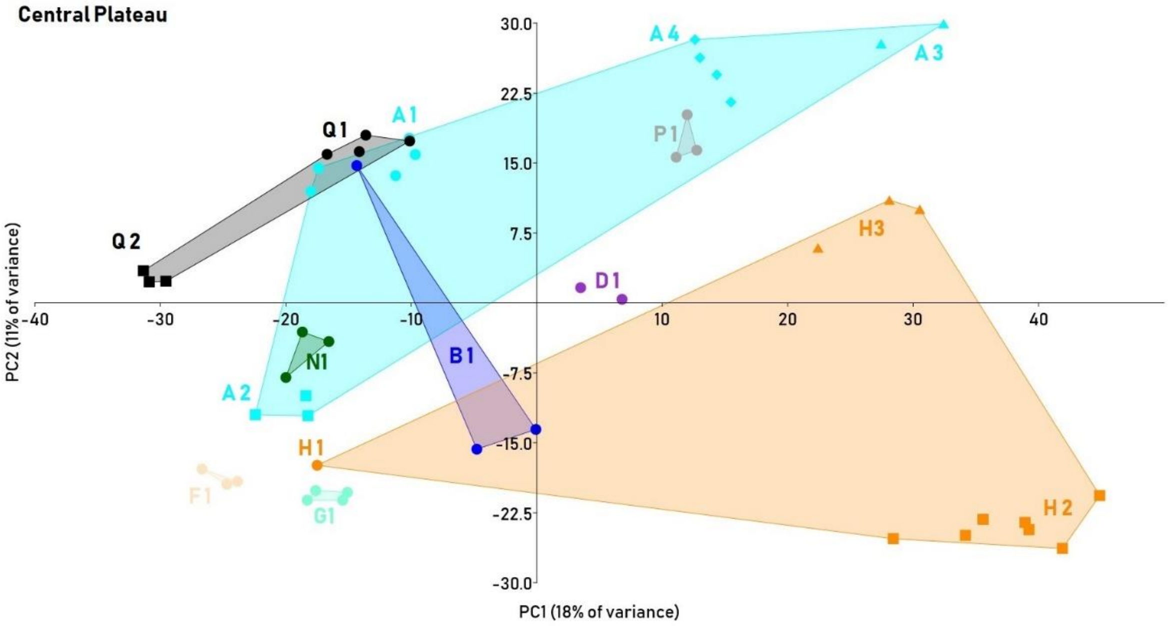

Bonferroni-corrected p values

	A	B	D	F	G	H	N	P	Q
A		1	1	0.043	0.022	0.004	0.104	0.432	0.004
B	1		1	1	1	0.464	1	1	0.288
D	1	1		1	1	0.986	1	1	1
F	0.043	1	1		1	0.126	1	1	0.335
G	0.022	1	1	1		0.007	1	1	0.090
H	0.004	0.464	0.986	0.126	0.007		0.108	0.079	0.007
N	0.104	1	1	1	1	0.108		1	0.335
P	0.432	1	1	1	1	0.079	1		0.342
Q	0.004	0.288	1	0.335	0.090	0.007	0.335	0.342	

Bonferroni-corrected p values litters of household A

	A1	A2	A3	A4
A1		0.116	0.292	0.058
A2	0.116		0.617	0.176
A3	0.292	0.617		0.394
A4	0.058	0.176	0.394	

Bonferroni-corrected p values litters of household H

	H1	H2	H3
H1		0.381	0.743
H2	0.381		0.025
H3	0.743	0.025	

Bonferroni-corrected p values litters of household Q

	Q1	Q2
Q1		0.032
Q2	0.032	

Supplementary Figure S7. Principal component analysis (PCA) of the bacterial skin microbiota of the puppies belonging to households of the Northern Alps bioregion. The difference between all groups was statistically significant by PERMANOVA (p = 0.0286, F = 5.52). Statistically significant p values are highlighted in yellow.

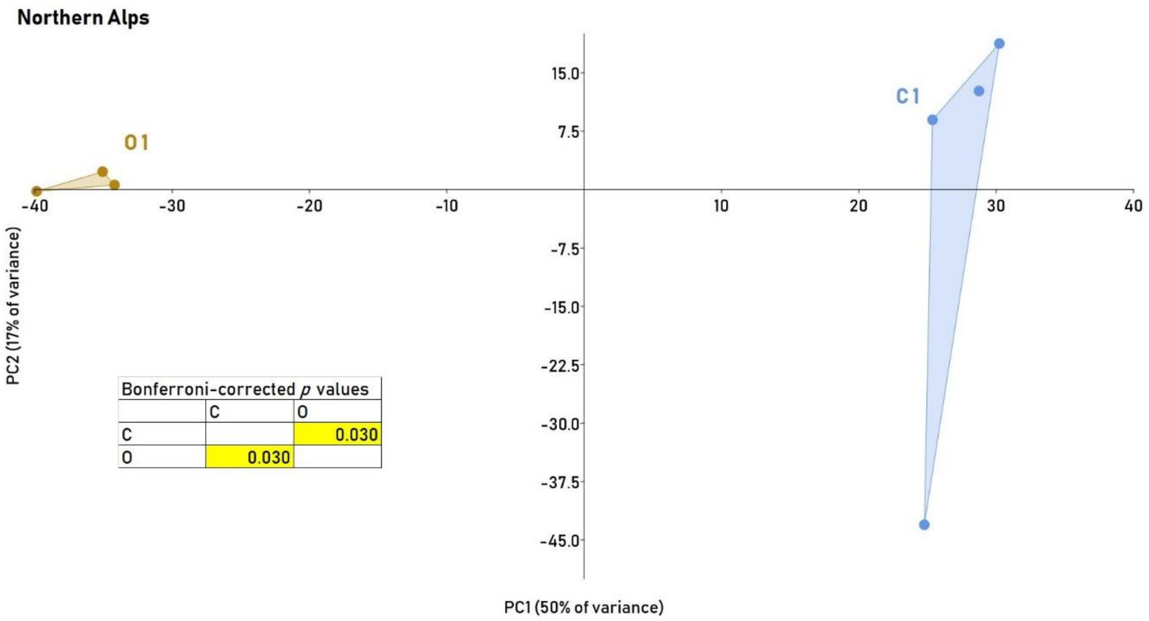

Bonferroni-corrected p values		
	C	O
C		0.030
O	0.030	

Supplementary Figure S8. Principal component analysis (PCA) of the bacterial skin microbiota of the puppies belonging to the household of the Jura bioregion. The difference between all groups was statistically significant by PERMANOVA (p = 0.0001, F = 4.28). No statistically significant difference was observed between the litters of this household.

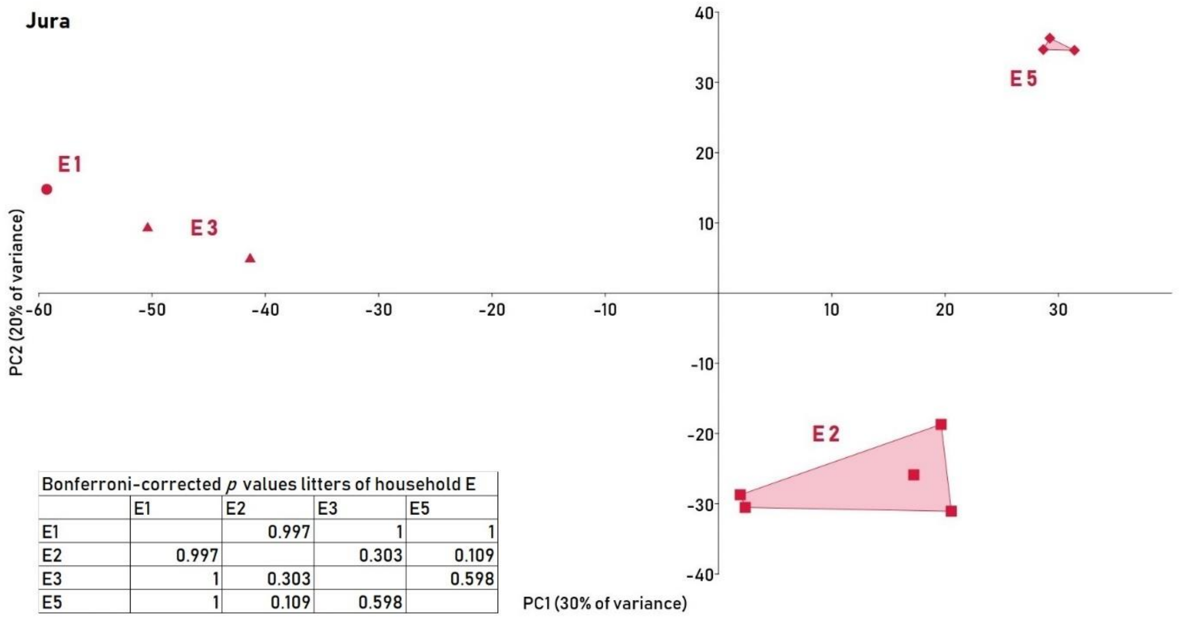

Bonferroni-corrected p values litters of household E				
	E1	E2	E3	E5
E1		0.997	1	1
E2	0.997		0.303	0.109
E3	1	0.303		0.598
E5	1	0.109	0.598	

Supplementary Figure S9. Principal component analysis (PCA) of the bacterial skin microbiota of the puppies from households in Hungary and France. The difference between all groups was statistically significant by PERMANOVA (p = 0.0001, F = 2.95). No statistically significant difference was observed between the litters of the French household (L) (p = 1, F = 0.37).

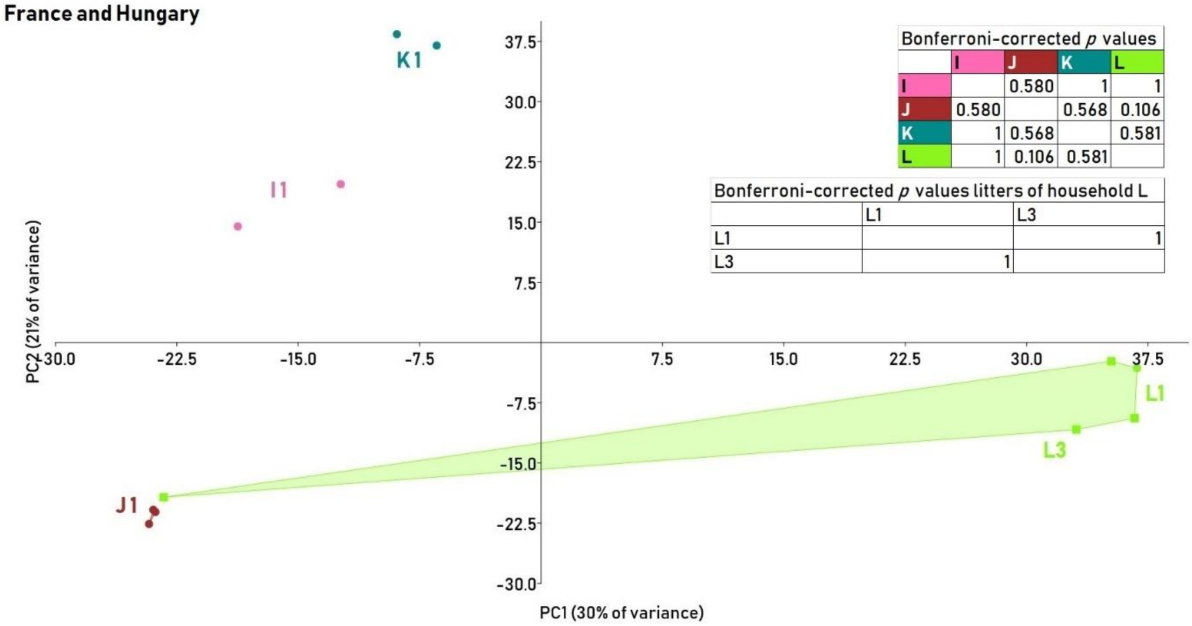

Supplementary Figure S10. Principal component analysis (PCA) of the cutaneous bacterial microbial profiles of the puppies grouped by season of sampling. The difference between all groups was significant by PERMANOVA (p = 0.0001, F = 3.54). The Bonferroni-corrected p values for the difference between pairs of groups are shown in the table below. Statistically significant p values are highlighted in yellow.

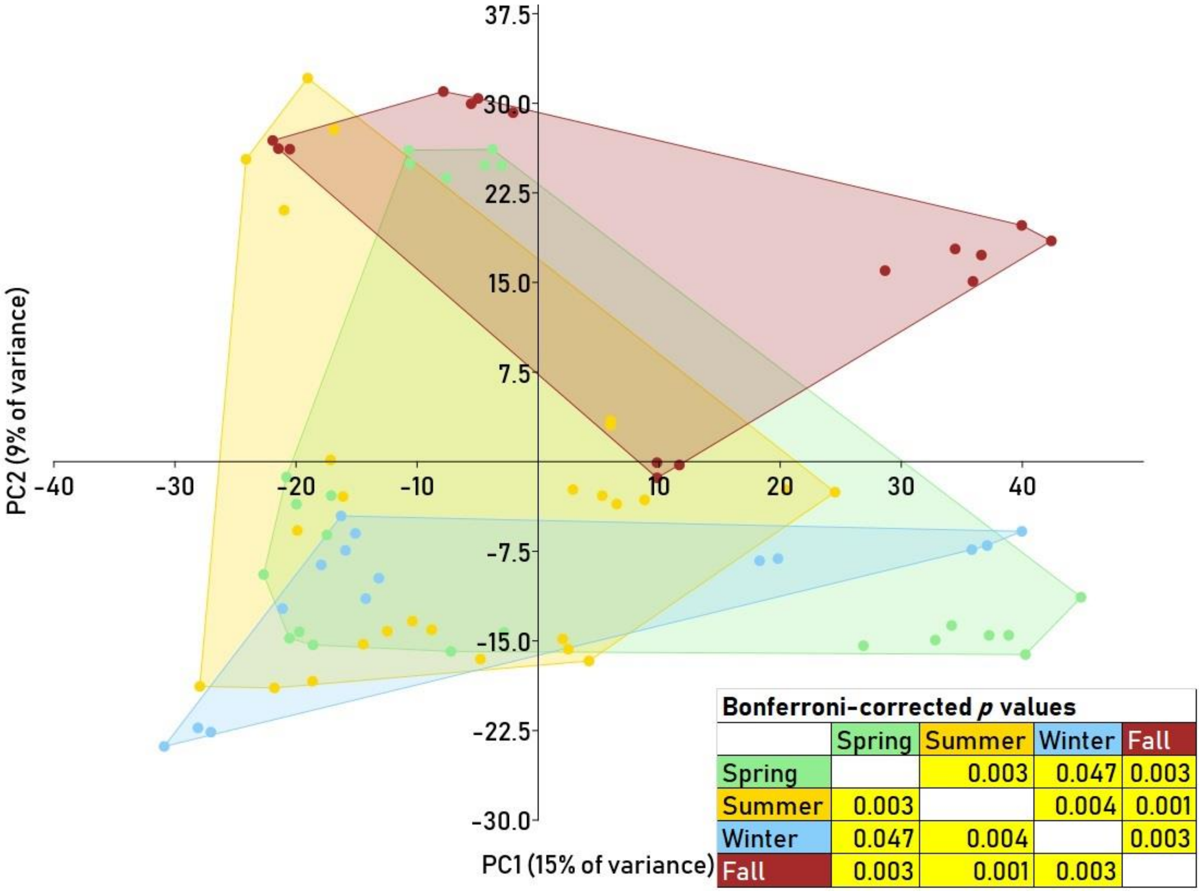

Bonferroni-corrected *p* values

	Spring	Summer	Winter	Fall
Spring		0.003	0.047	0.003
Summer	0.003		0.004	0.001
Winter	0.047	0.004		0.003
Fall	0.003	0.001	0.003	

Supplementary Figure S11. Visual comparison by principal component analysis (PCA) of the cutaneous (gray) and oral (black) mycobiota of the puppies (A) and adult dogs (B); PERMANOVA *p* and *F* values are shown.

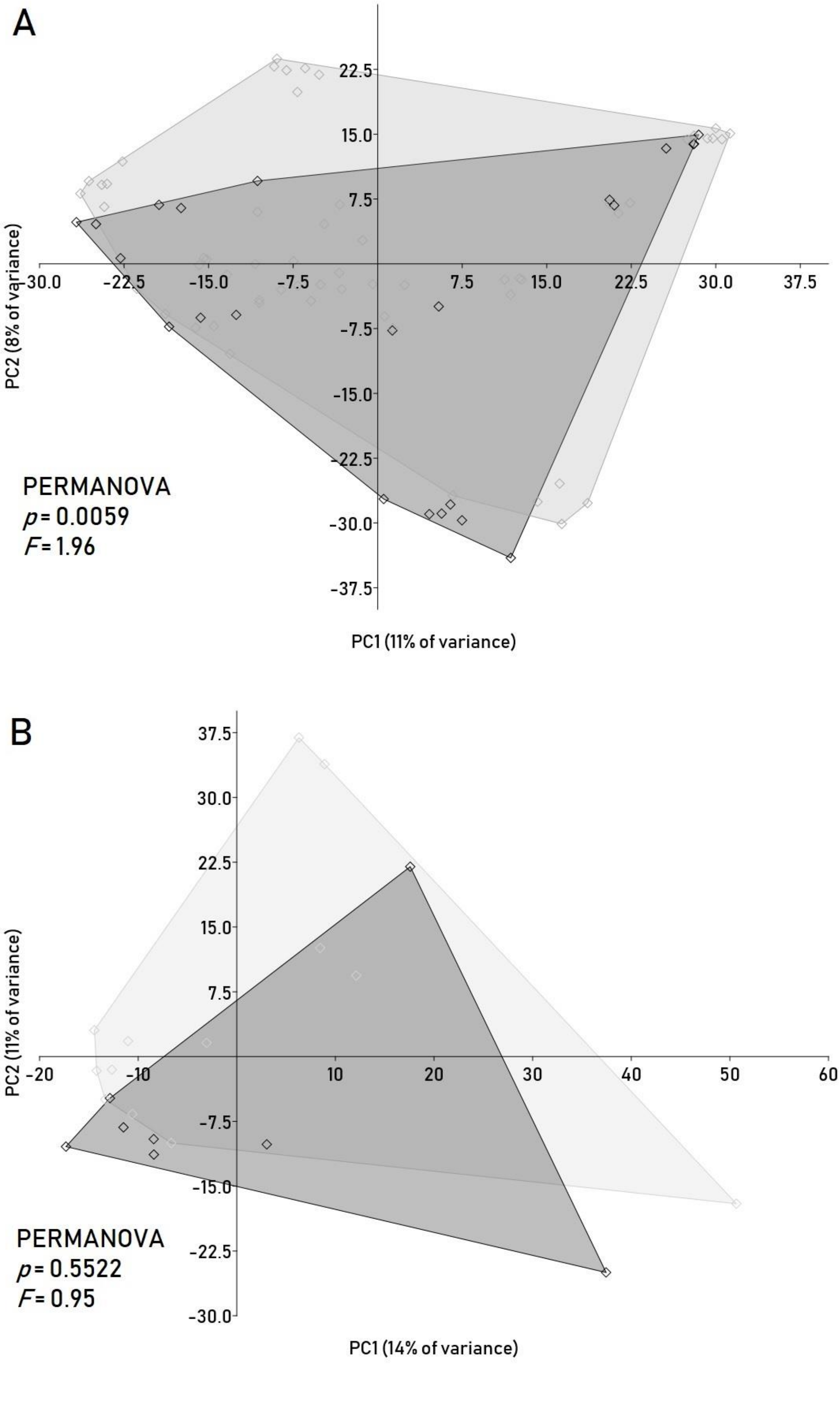

Supplementary Figure S12. Principal component analysis (PCA) of the skin mycobiota of the puppies according to geographical origin. The difference between all groups was significant by PERMANOVA (p = 0.0001, F = 2.69). Statistically significant Bonferroni corrected p values are highlighted in yellow.

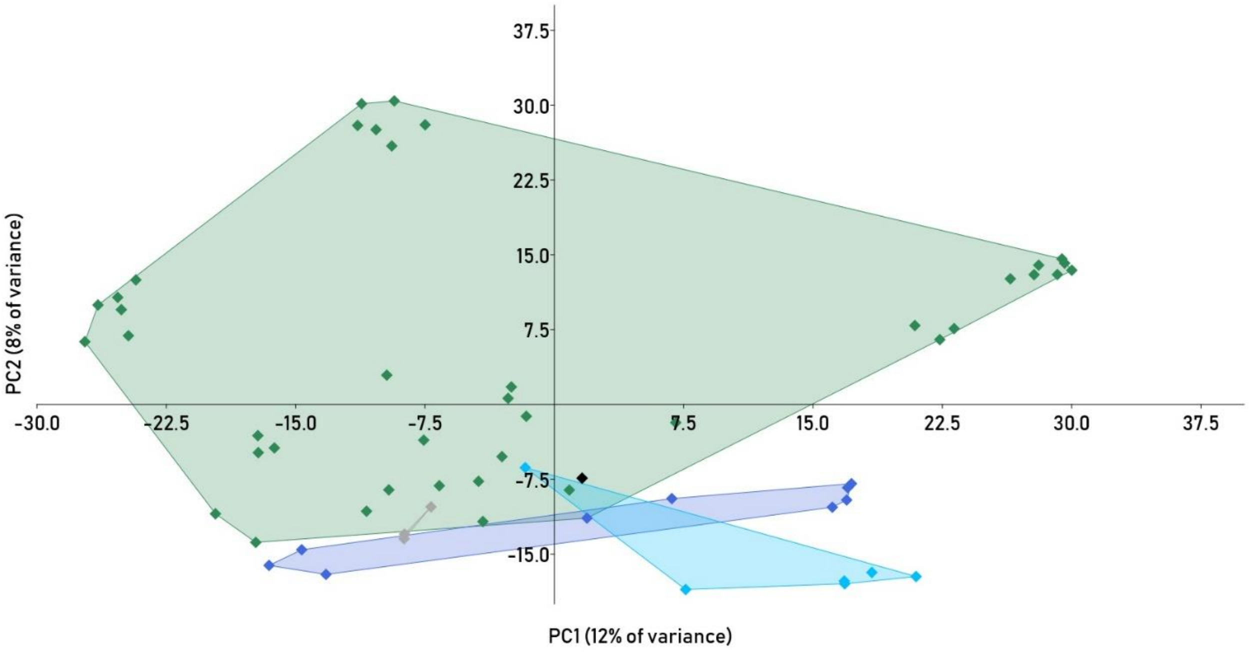

Bonferroni-corrected *p* values					
	CentralPlateau	NorthernAlps	Jura	Hungary	France
CentralPlateau		0.001	0.001	0.985	0.006
NorthernAlps	0.001		0.004	1	0.122
Jura	0.001	0.004		1	0.12
Hungary	0.985	1	1		1
France	0.006	0.122	0.12	1	

Supplementary figure S13. Principal component analysis (PCA) of the fungal skin microbiota of the puppies according to owners. The difference between all groups was significant by PERMANOVA (p = 0.0001, F = 4.70), and between seven pairs of groups as indicated by Bonferroni-corrected p values shown in the table below. Statistically significant p values are highlighted in yellow.

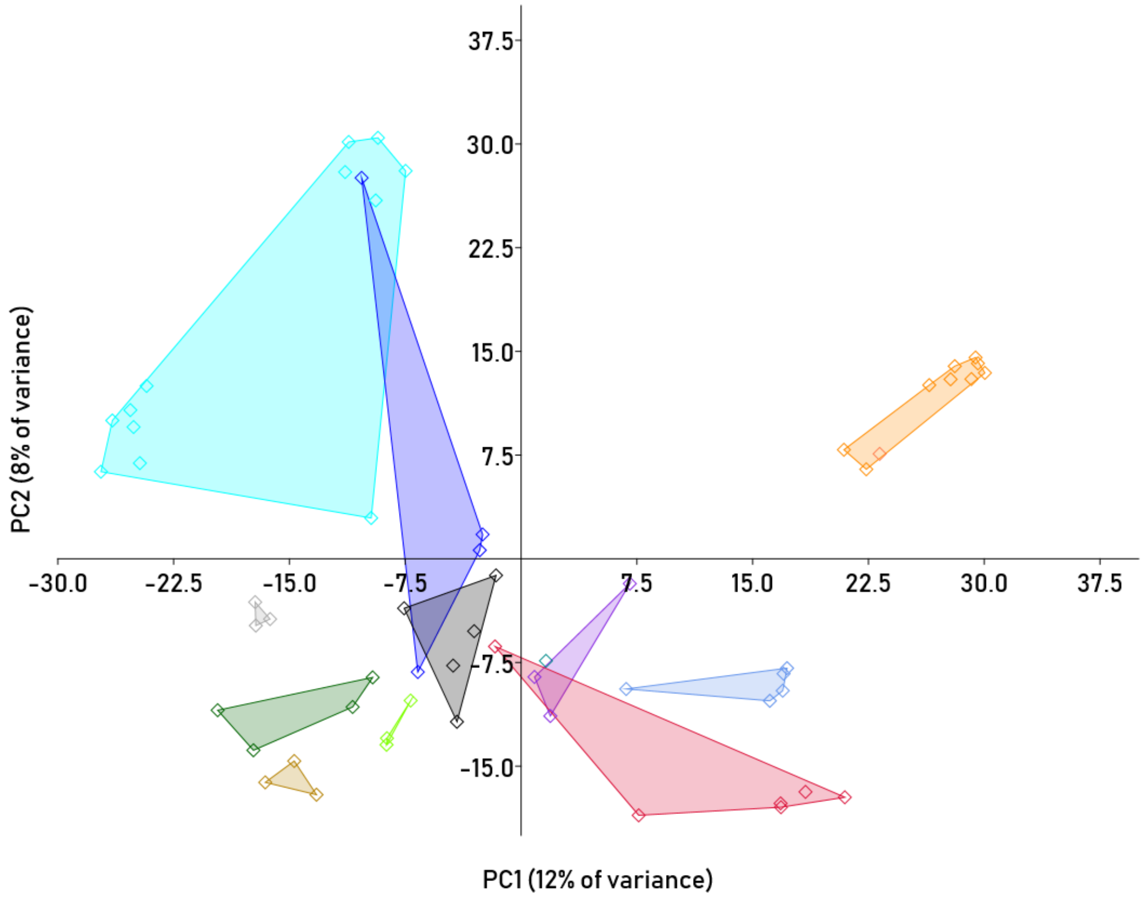

Bonferroni-corrected p values

	A	B	C	D	E	H	K	L	N	O	P	Q
A		1	0.020	0.158	0.007	0.007	1	0.145	0.033	0.112	0.172	0.013
B	1		0.508	1	0.350	0.086	1	1	1	1	1	0.541
C	0.020	0.508		1	0.172	0.040	1	1	0.607	1	1	0.535
D	0.158	1	1		0.818	0.218	1	1	1	1	1	1
E	0.007	0.350	0.172	0.818		0.020	1	0.785	0.297	0.805	0.825	0.277
H	0.007	0.086	0.040	0.218	0.020		1	0.244	0.040	0.271	0.238	0.026
K	1	1	1	1	1	1		1	1	1	1	1
L	0.145	1	1	1	0.785	0.244	1		1	1	1	1
N	0.033	1	0.607	1	0.297	0.040	1	1		1	1	0.515
O	0.112	1	1	1	0.805	0.271	1	1	1		1	1
P	0.172	1	1	1	0.825	0.238	1	1	1	1		1
Q	0.013	0.541	0.535	1	0.277	0.026	1	1	0.515	1	1	

Supplementary Figure S14. Principal component analysis (PCA) of the skin mycobiota of the puppies belonging to households of the Central Plateau bioregion. The difference between all groups was statistically significant by PERMANOVA (p = 0.0001, F = 5.07). The differences between different litters from the same household (A, H and Q) was statistically significant by PERMANOVA for litters in households A (p = 0.0002, F = 4.13) and H (p = 0.0092, F = 6.45), but not for litters in household Q. Statistically significant Bonferroni-corrected p values between pairs of households and litters are highlighted in yellow.

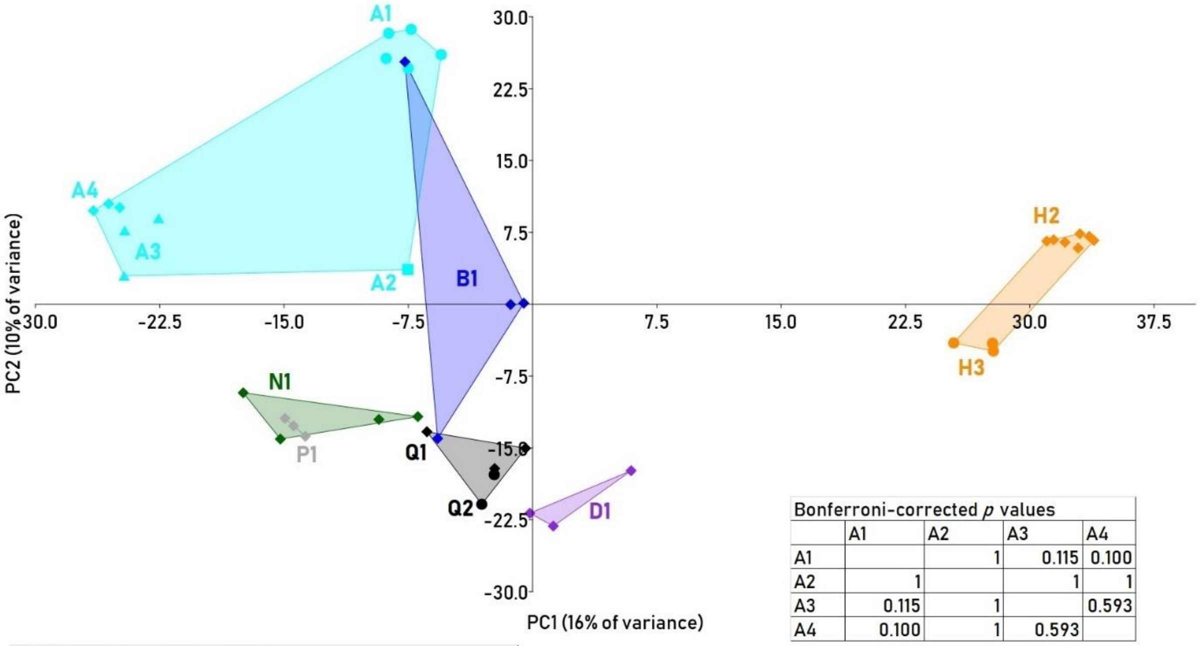

Bonferroni-corrected p values

	A	B	Q	D	P	N	H
A		0.439	0.006	0.050	0.044	0.013	0.002
B	0.439		0.132	0.599	1	0.561	0.017
Q	0.006	0.132		0.420	0.359	0.160	0.006
D	0.050	0.599	0.420		1	0.643	0.067
P	0.044	1	0.359	1		0.529	0.063
N	0.013	0.561	0.160	0.643	0.529		0.019
H	0.002	0.017	0.006	0.067	0.063	0.019	

Bonferroni-corrected p values

	A1	A2	A3	A4
A1		1	0.115	0.100
A2	1		1	1
A3	0.115	1		0.593
A4	0.100	1	0.593	

Bonferroni-corrected p values

	H2	H3
H2		0.008
H3	0.008	

Bonferroni-corrected p values

	Q2	Q1
Q2		0.102
Q1	0.102	

Supplementary Figure S15. Principal component analysis (PCA) of the skin mycobiota of the puppies from households in the bioregion Northern Alps, Hungary and France. The difference between all groups was statistically significant by PERMANOVA (*p* = 0.0001, *F* = 3.7). No statistically significant difference was observed between the litters of the French household (L) (*p* = 1, *F* = 0.849).

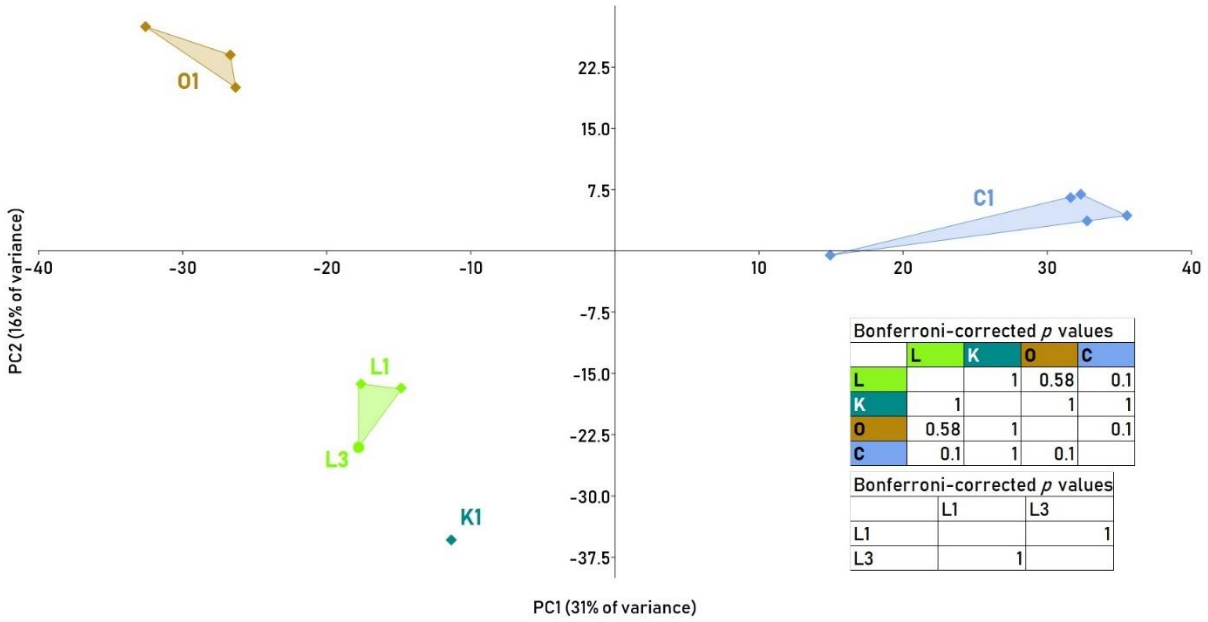

Supplementary Figure S16. Principal component analysis (PCA) of the bacterial mycobiota of the puppies belonging to the household of the Jura bioregion. The difference between all groups was statistically significant by PERMANOVA (*p* = 0.0164, *F* = 3.48). No statistically significant difference was observed between the litters of this household.

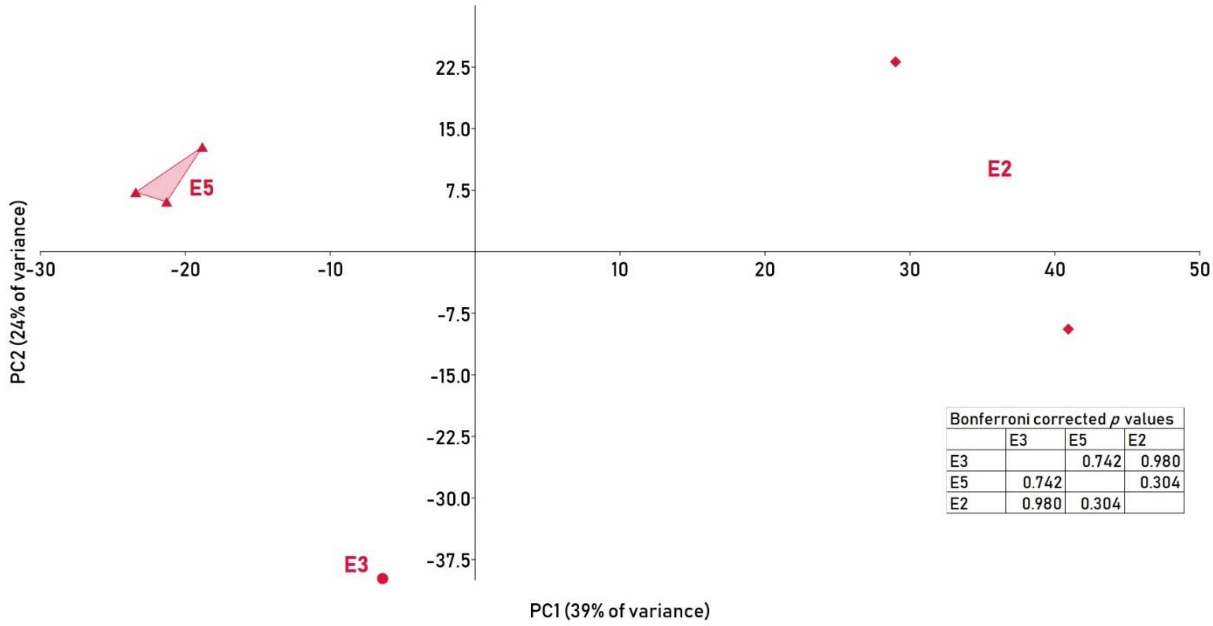

Supplementary Figure S17. Principal component analysis (PCA) of the cutaneous fungal microbial profiles of the puppies grouped by season of sampling. The difference between all groups was significant by PERMANOVA (p = 0.0001, F = 3.22). The Bonferroni-corrected p values for the difference between pairs of groups are shown in the table below, statistically significant p values are highlighted in yellow.

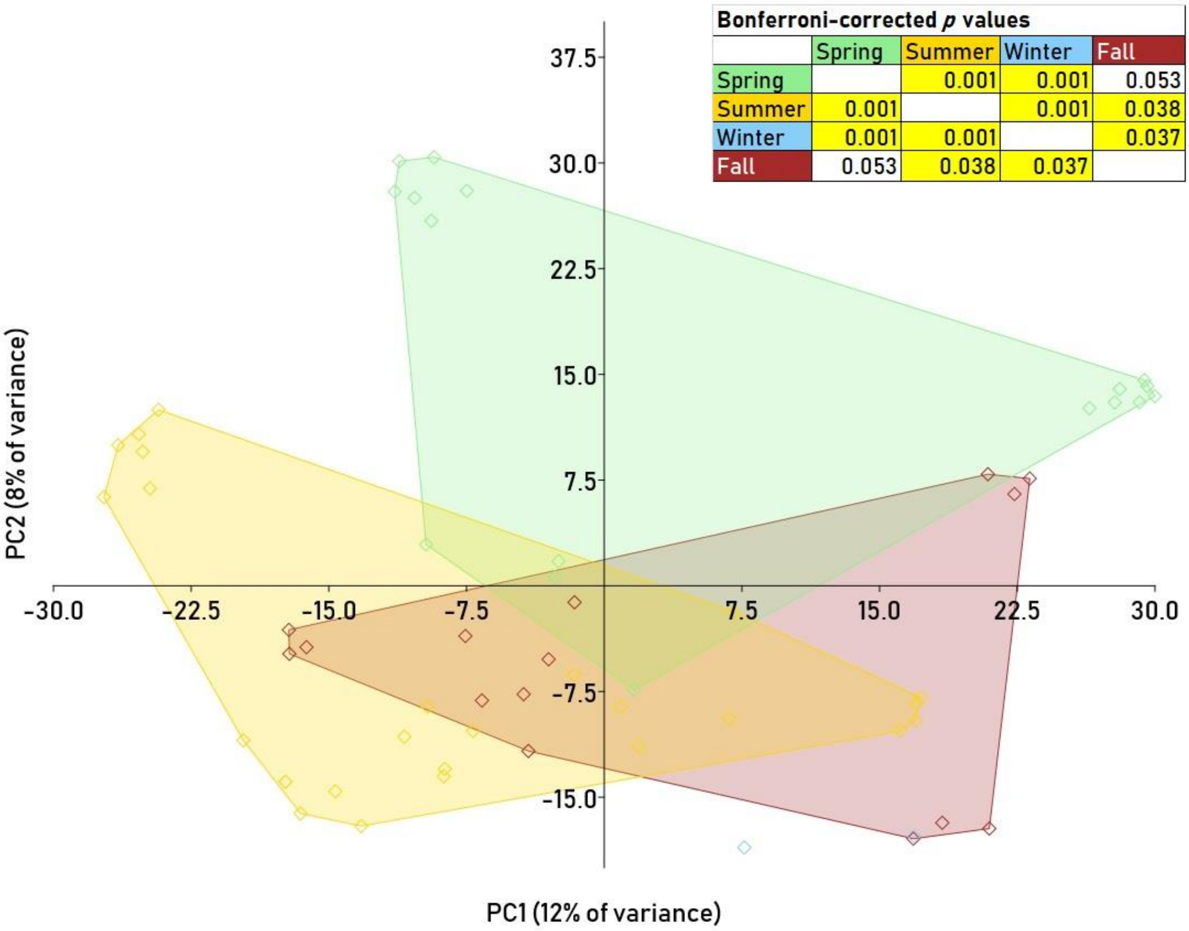

Bonferroni-corrected *p* values

	Spring	Summer	Winter	Fall
Spring		0.001	0.001	0.053
Summer	0.001		0.001	0.038
Winter	0.001	0.001		0.037
Fall	0.053	0.038	0.037	

Supplementary figure S18. Scatter plots including Pearson correlation coefficients *r* and *p* values of the comparison of the number of observed OTUs (**A**), Chao1 (**B**), Shannon diversity indices (**C**), and of the bacterial and fungal cutaneous microbial profiles of the puppies.

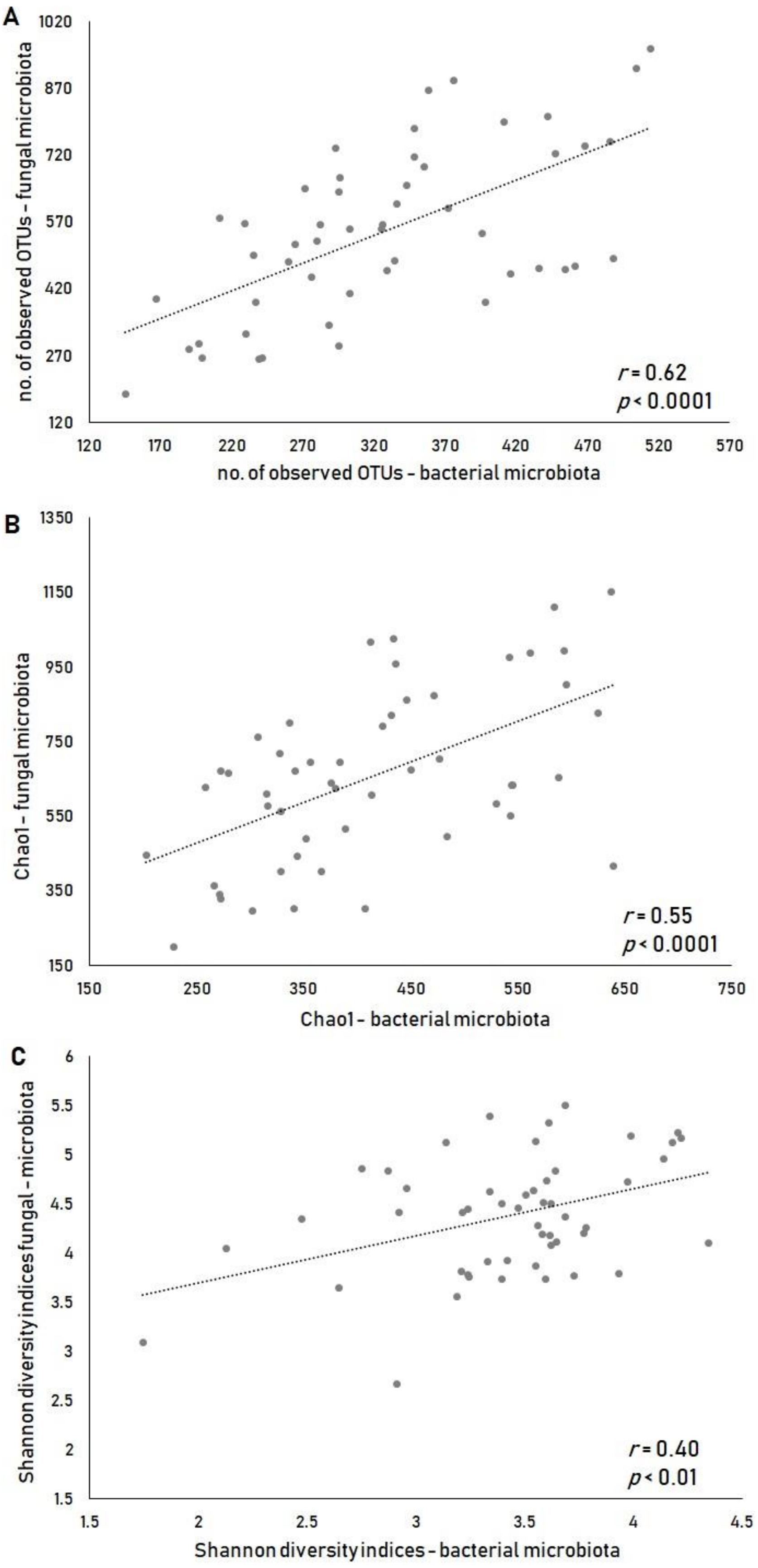

Supplementary Tables

Supplementary table S1. Average (AM) and standard error of the mean (SEM) for the α-diversity estimators number of observed number of OTUs (No. OTUs), Chao1 and Shannon diversity index (H) for the cutaneous bacterial microbiota of the puppies grouped by the hygiene status of the respective owners (1=low standard, 2=average standard, 3=high standard) and grouped by the access to the outdoor environment (1=inside, 2=inside with access to outdoor environment, 3=outside). The statistical difference of those estimators between groups was analyzed by two-sample t-test/Wilcoxon Mann–Whitney test (normally/non-normally distributed data)

Estimator	Hygiene						Outdoor environment					
	Variable	AM	SEM	*p* 1 and 2	*p* 1 and 3	*p* 2 and 3	Variable	AM	SEM	*p* 1 and 2	*p* 1 and 3	*p* 2 and 3
	1	396.6	40.9				1	391.9	42.8			
No. OTUs	2	468.5	37.8	0.8659	0.3531	1	2	452.3	33.1	1	0.1740	0.3983
	3	498.5	38.3				3	526.9	42.1			
	1	469.7	48.9				1	481.8	54.5			
Chao1	2	570.0	46.0	1	0.3206	0.4616	2	533.9	38.6	1	0.3206	0.4616
	3	585.7	45.8				3	626.8	52.1			
	1	4.0	0.2				1	4.1	0.2			
H	2	4.0	0.1	0.7294	0.4204	1	2	4.1	0.1	1	1	1
	3	4.4	0.1				3	4.3	0.1			

Supplementary table S2. Average (AM) and standard error of the mean (SEM) for the α-diversity estimators number of observed number of OTUs (No. OTUs), Chao1 and Shannon diversity index (H) for the cutaneous fungal microbiota of the puppies grouped by the hygiene status of the respective owners (1=low standard, 2=average standard, 3=high standard) and grouped by the access to the outdoor environment (1=inside, 2=inside with access to outdoor environment, 3=outside). The statistical difference of those estimators between groups was analyzed by two-sample t-test/Wilcoxon Mann–Whitney test (normally/non-normally distributed data)

Estimator	Hygiene						Outdoor environment					
	**Variable**	**AM**	**SEM**	***p* 1 and 2**	***p* 1 and 3**	***p* 2 and 3**	**Variable**	**AM**	**SEM**	***p* 1 and 2**	***p* 1 and 3**	***p* 2 and 3**
No. OTUs	1	310.4	31.3	0.8979	0.7766	0.7511	1	277.7	29.2	0.3112	1	0.6146
	2	321.1	22.0				2	341.5	22.9			
	3	302.1	21.7				3	300.8	20.7			
Chao1	1	380.9	36.8	0.3949	1	1	1	344.2	35.3	0.3949	1	1
	2	414.0	28.5				2	420.0	28.6			
	3	375.1	25.6				3	386.3	25.4			
H	1	3.2	0.1	0.0064	0.2441	0.1586	1	3.0	0.2	0.8520	0.1277	0.1676
	2	3.6	0.1				2	3.3	0.1			
	3	3.3	0.1				3	3.5	0.1			

Supplementary table S3. Overview of one-way permutational multivariate analysis of variance (PERMANOVA, 9999 permutations) with Bray-Curtis similarity distance performed with the Paleontological Statistics (PAST version 3.12) software and with unweighted and weighted Unifrac distance performed in R v.3.6.0 67 using the packages phyloseq (v.1.28.0) and vegan (v2.5-5).

		**PERMANOVA (9999 permutations) using distance:**					
**Comparisons**	**Variables**	**Bray Curtis**		**Unweighted Unifrac**		**Weighted Unifrac**	
		F	***P* value**	***F***	***P* value**	***F***	***P* value**
by sample site	Adult dogs: skin vs. oral	16.27	0.0001	15.67	0.0001	31.21	0.0001
	Puppies: skin vs. oral	31.34	0.0001	23.07	0.0001	42.46	0.0001
by age group	Skin: puppies vs. adult	3.91	0.0002	3.04	0.0008	3.78	0.0044
	Oral: puppies vs. adult	24.04	0.0001	17.84	0.0001	29.82	0.0001
for skin of puppies only	Bioregions	3.41	0.0001	2.57	0.0001	3.78	0.0001
	Sex	0.67	0.8879	0.71	0.8825	0.51	0.8606
	Hygiene	3.34	0.0001	2.69	0.0001	3.16	0.0024
	Environment	3.50	0.0001	2.71	0.0001	4.51	0.0003
	Litter	6.63	0.0001	3.82	0.0001	11.12	0.0001
	household	4.59	0.0001	3.03	0.0001	5.66	0.0001
	allergic (yes/no)	1.01	0.3896	1.05	0.3249	1.34	0.2004

Supplementary table S4. Average (AM) and standard error of the mean (SEM) for the α-diversity estimators number of observed number of OTUs (No. OTUs), Chao1 and Shannon diversity index (H) for the cutaneous fungal microbiota of the puppies grouped by season of sampling. The statistical difference of those estimators between groups was analyzed by two-sample t-test/Wilcoxon Mann–Whitney test (normally/non-normally distributed data).

**Estimator**	**Season**	**AM**	**SEM**	***p* spring/summer**	***p* spring/winter**	***p* spring/fall**	***p* summer/fall**	***p* summer/winter**	***p* winter/fall**
No. OTUs	Spring	332.4	24.3	0.1941	1	1	0.0785	1	1
	Summer	263.3	18.7						
	Winter	293.5	82.5						
	Fall	366.1	27.3						
Chao1	Spring	413.2	29.2	0.4822	1	1	0.2848	1	1
	Summer	340.5	23.8						
	Winter	355.8	77.2						
	Fall	450.1	26.1						
H	Spring	3.4	0.1	1	0.1600	1	0.3279	0.3636	1
	Summer	3.1	0.1						
	Winter	4.1	0.1						
	Fall	3.6	0.1						
